# Telephone-Based Guideline-Directed Medical Therapy Optimization in Navajo Nation: The Hózhó Randomized Clinical Trial

**DOI:** 10.1001/jamainternmed.2024.1523

**Published:** 2024-04-07

**Authors:** Lauren A. Eberly, Ada Tennison, Daniel Mays, Chih-Yuan Hsu, Chih-Ting Yang, Ernest Benally, Harriett Beyuka, Benjamin Feliciano, C. Jane Norman, Maria Ynes Brueckner, Clybert Bowannie, Daniel R. Schwartz, Erica Lindsey, Stephen Friedman, Elizabeth Ketner, Pamela Detsoi-Smiley, Yu Shyr, Sonya Shin, Maricruz Merino

**Affiliations:** 1 Gallup Indian Medical Center, Indian Health Service, Gallup, New Mexico; 2 Division of Cardiovascular Medicine, Department of Medicine, Hospital of the University of Pennsylvania, Philadelphia; 3 Penn Cardiovascular Outcomes, Quality, and Evaluative Research Center, Cardiovascular Institute, University of Pennsylvania, Philadelphia; 4 Penn Cardiovascular Center for Health Equity and Social Justice, University of Pennsylvania, Philadelphia; 5 Leonard Davis Institute of Health Economics, University of Pennsylvania, Philadelphia; 6 Department of Biostatistics, Vanderbilt University Medical Center, Nashville, Tennessee; 7 Office of Quality, Division of Innovations and Improvement, Indian Health Service Headquarters, Rockville, Maryland; 8 Corporal Michael J. Crescenz Veterans Affairs Medical Center, Philadelphia, Pennsylvania; 9 Department of Global Health and Social Medicine, Harvard Medical School, Boston, Massachusetts; 10 Division of Global Health Equity, Brigham and Women’s Hospital, Boston, Massachusetts

## Abstract

**Importance:**

Underutilization of guideline-directed medical therapy for heart failure with reduced ejection fraction is a major cause of poor outcomes. For many American Indian patients receiving care through the Indian Health Service, access to care, especially cardiology care, is limited, contributing to poor uptake of recommended therapy.

**Objective:**

To examine whether a telehealth model in which guideline-directed medical therapy is initiated and titrated over the phone with remote telemonitoring using a home blood pressure cuff improves guideline-directed medical therapy use (eg, drug classes and dosage) in patients with heart failure with reduced ejection fraction in Navajo Nation.

**Design, Setting, and Participants:**

The Heart Failure Optimization at Home to Improve Outcomes (Hózhó) randomized clinical trial was a stepped-wedge, pragmatic comparative effectiveness trial conducted from February to August 2023. Patients 18 years and older with a diagnosis of heart failure with reduced ejection fraction receiving care at 2 Indian Health Service facilities in rural Navajo Nation (defined as having primary care physician with 1 clinical visit and 1 prescription filled in the last 12 months) were enrolled. Patients were randomized to the telehealth care model or usual care in a stepped-wedge fashion, with 5 time points (30-day intervals) until all patients crossed over into the intervention. Data analyses were completed in January 2024.

**Intervention:**

A phone-based telehealth model in which guideline-directed medical therapy is initiated and titrated at home, using remote telemonitoring with a home blood pressure cuff.

**Main Outcomes and Measures:**

The primary outcome was an increase in the number of guideline-directed classes of drugs filled from the pharmacy at 30 days postrandomization.

**Results:**

Of 103 enrolled American Indian patients, 42 (40.8%) were female, and the median (IQR) age was 65 (53-77) years. The median (IQR) left ventricular ejection fraction was 32% (24%-36%). The primary outcome occurred significantly more in the intervention group (66.2% vs 13.1%), thus increasing uptake of guideline-directed classes of drugs by 53% (odds ratio, 12.99; 95% CI, 6.87-24.53; *P* < .001). The number of patients needed to receive the telehealth intervention to result in an increase of guideline-directed drug classes was 1.88.

**Conclusions and Relevance:**

In this heart failure trial in Navajo Nation, a telephone-based strategy of remote initiation and titration for outpatients with heart failure with reduced ejection fraction led to improved rates of guideline-directed medical therapy at 30 days compared with usual care. This low-cost strategy could be expanded to other rural settings where access to care is limited.

**Trial Registration:**

ClinicalTrials.gov Identifier: NCT05792085

## Key Points

**Question:**

In settings with limited access to care, does a phone-based telehealth model for heart failure with reduced ejection fraction increase uptake of guideline-directed medical therapy?

**Findings:**

In this stepped-wedge, pragmatic randomized clinical trial including 103 American Indian patients, a phone-based telehealth model led to higher rates of guideline-directed classes of drugs filled from the pharmacy at 30 days (66.2% vs 13.1%), a significant difference.

**Meaning:**

A low-cost strategy of phone-based guideline-directed drug optimization can improve guideline-directed medical therapy rates in settings where access to care is limited.

## Teaser

This stepped-wedge, pragmatic randomized clinical trial examines whether a telehealth model in which guideline-directed medical therapy is initiated and titrated over the phone with remote telemonitoring using a home blood pressure cuff improves guideline-directed medical therapy use in patients with heart failure with reduced ejection fraction in Navajo Nation.

## Introduction

Four-pillar pharmacotherapy has now been established as first-line treatment for patients with heart failure with reduced ejection fraction (HFrEF) given its clear benefit to reduce mortality, prevent HF hospitalizations, and improve quality of life.^1,2,3,4,5,6,7^ These 4 therapies include β-blockers, renin-angiotensin-aldosterone system (RAAS) inhibitors (angiotensin-converting enzyme inhibitors [ACEis], angiotensin receptor blockers [ARBs], or preferably angiotensin receptor–neprilysin inhibitors [ARNIs]), mineralocorticoid receptor antagonists (MRAs), and sodium-glucose cotransporter-2 inhibitors (SGLT2i). Despite strong evidence, real-world uptake of these therapies remains suboptimal.^8,9,10^ Underutilization of guideline-directed medical therapy (GDMT) for HFrEF is a major cause of poor outcomes.^11,12^

Many efforts to improve uptake of GDMT have had limited benefit or have relied on patients being seen in a health care facility.^13,14,15,16^ Additionally, there has been a lack of efforts designed specifically for racially marginalized patient groups, particularly American Indian patients. For many American Indian patients receiving care through the Indian Health Service (IHS), access to care, especially cardiology care, is limited.^17,18^ For American Indian patients living rurally on reservations, there are particular care access challenges.^19^ Given this, we designed a telehealth HFrEF model in rural Navajo Nation in which GDMT is initiated and titrated by phone with remote telemonitoring using a home blood pressure (BP) cuff. Phone-based GDMT optimization, if effective, is a low-cost, scalable intervention for resource-limited settings, especially where cardiology access is limited. The Heart Failure Optimization at Home to Improve Outcomes (Hózhó) randomized clinical trial was designed to test the hypothesis that phone-based GDMT optimization would lead to higher rates of GDMT utilization compared with usual care.

## Methods

### Trial Design Overview and Procedures

**Figure 1.**
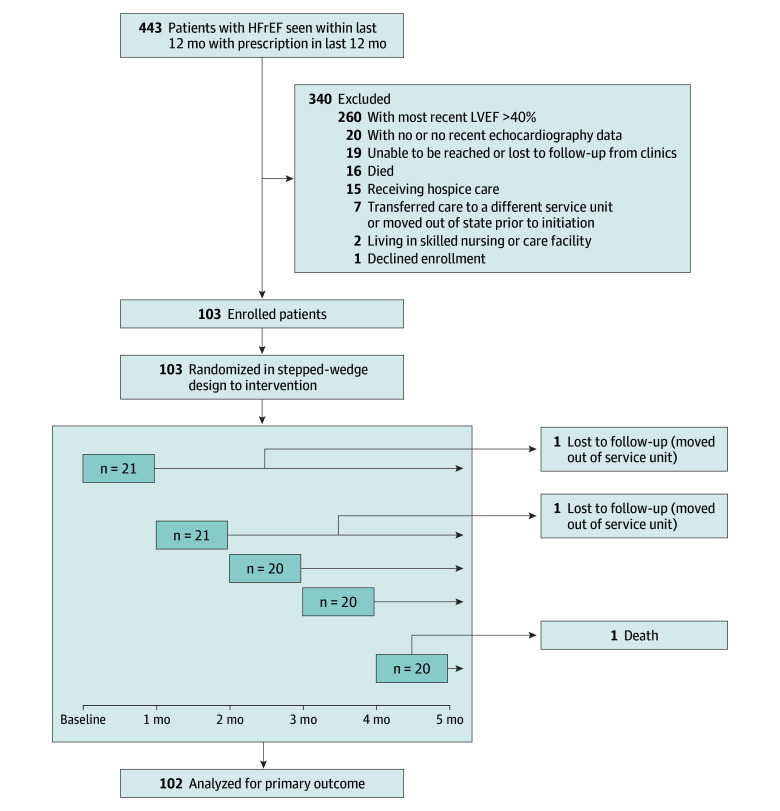
Study Design and CONSORT Diagram The Heart Failure Optimization at Home to Improve Outcomes (Hózhó) trial was a stepped-wedge randomized clinical trial. Patients were randomized to the telehealth intervention at 5 different time points (at 30-day intervals) until all patients had crossed over into the intervention arm of telehealth care. The trial examined whether a telehealth (phone-based) guideline-directed medical therapy optimization program for outpatients with heart failure with reduced ejection fraction (HFrEF) led to higher rates of guideline-directed medical therapy at 30 days postrandomization compared with usual care. LVEF indicates left ventricular ejection fraction.

The full study protocol is accessible through the study website^20^ and is available in Supplement 1. *Hózhó* is a Diné (Navajo) concept that captures the philosophy of health, balance, and wellness.^21^ This study used a closed-cohort stepped-wedge cluster-randomized design.^22^ All enrolled patients were randomized to the telehealth care model or usual care in a stepped-wedge fashion, with 5 sequential time points at 30-day intervals, until all patients had crossed over into the intervention (ie, cluster 1 had immediate implementation of telehealth model while clusters 2 to 5 remained in usual care; at 30 days, cluster 2 crossed over; at 60 days, cluster 3 crossed over; and so on) (Figure 1). Patients from each site were randomized to each cluster. Patients and clinicians were not blinded, but study staff who assessed and analyzed outcomes were blinded to patient cluster and treatment assignment. A stepped-wedge design was selected to facilitate rollout and ensure all patients ultimately received the intervention, given questionable equipoise and stakeholder preference. Although this was part of an IHS quality improvement program, all patients provided verbal informed consent by phone prior to enrollment. This study began on January 5, 2023, and enrollment was completed by February 1, 2023. This trial was conducted in accordance with the principles of the Declaration of Helsinki and was approved by the Navajo Nation Human Research Review Board (NNR23.470). We followed the Consolidated Standards of Reporting Trials (CONSORT) reporting guideline.

### Study Participants

Inclusion criteria included age of 18 years or older, *International Statistical Classification of Diseases and Related Health Problems, Tenth Revision *code I50*, left ventricular EF of 40% or less, a primary care physician (PCP) and clinical encounter at one of the 2 IHS sites in the last 12 months, and a prescription in the last 12 months (Figure 1). Medical records were reviewed to confirm left ventricular EF of 40% or less on the most recent echocardiogram. Patients receiving hospice care, not living at home (eg, at a skilled nursing or acute rehabilitation facility), or who declined participation were excluded. Eligible patients were contacted by phone, consented, and enrolled if they were still living in the IHS service unit.

### Trial Setting

This trial was performed at 2 IHS ambulatory clinic sites in Navajo Nation, one of which is a large IHS site serving as a major referral hospital for Navajo Nation and the other is a smaller, even more rural IHS site^23^ (eFigure 1 in Supplement 2). Both clinics are located in rural eastern Navajo Nation, where cardiology care is limited. HF care is provided primarily by PCPs, but echocardiography is available at the larger IHS site. Referrals for specialty care can be made to facilities 2 to 3 hours away, with a median referral time of 6 months.^24^ Medications are provided free of charge for enrolled IHS members, including sacubitril-valsartan and empagliflozin.

### Intervention

#### Teleheath Model Design

**Figure 2.**
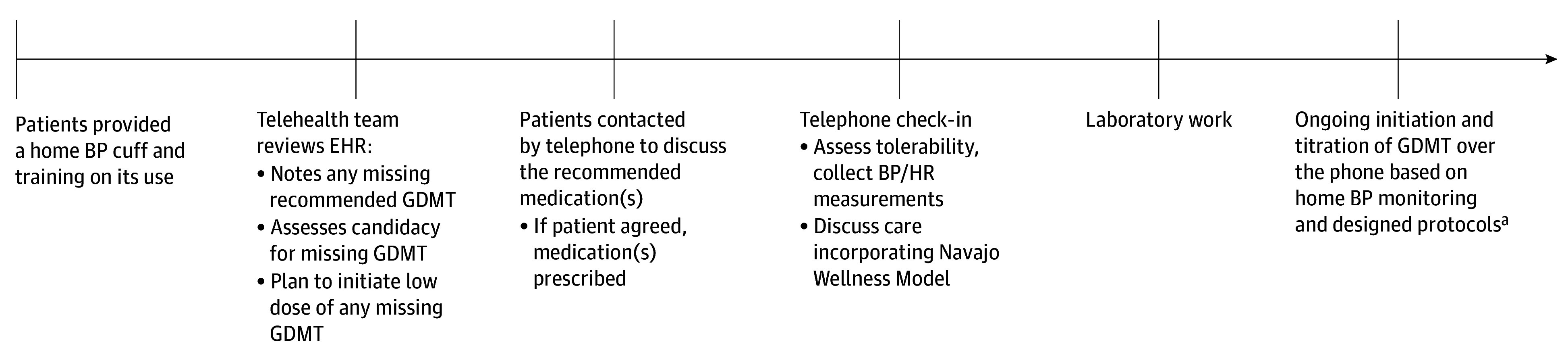
Overview of Telehealth Model In this telehealth model, (1) patients were provided with a home blood pressure (BP) cuff and training on its use in their preferred language (ie, Navajo or English). (2) The electronic health record (EHR) was evaluated by the telehealth team to identify missing guideline-directed medical therapy (GDMT) therapy and assess eligibility for each missing therapy. If a patient was deemed eligible for missing therapy or therapies, these were prescribed by the telehealth team, with guidance provided by designed protocols (eFigure 2 in Supplement 2) in line with updated American College of Cardiology/American Heart Association/Heart Failure Society of America guidelines. (3) Patients were contacted by the telehealth team to discuss medication(s) recommendations and therapy prescribed if patient agreed; laboratory work was ordered and patients were instructed to come in for blood work 1 to 2 weeks after every medication initiation or titration (except for β-blockers) in-person at the facility, as recommended by the telehealth protocols. (4) A check-in phone call occurred after 1 week to assess tolerability, collect home blood pressure and heart rate (HR) recordings, and remind patients to get laboratory work if they had not yet had laboratory testing performed as instructed. (5) Patients were called 1 week later to continue medication initiation and titration as outlined in the protocols, with the goal to get patients taking all eligible therapies within 30 days. Ongoing calls occurred every 1 to 2 weeks for continued medication titration until patients were taking maximally tolerated doses of GDMT. ^a^Goal to receive quadruple therapy (or all eligible therapies) by 30 days.

A telehealth model for HFrEF was designed as part of an IHS Office of Quality Innovations Award to improve care (Figure 2). We previously determined that major clinician-level barriers to GDMT uptake were clinical burden, time constraints, limited clinic visit availability, lack of comfort with newer therapies, and clinical guidelines; the main patient-level barriers were lack of transportation and clinician availability.^25^ We worked with community advisers to design a telehealth model to address these barriers. Given limited broadband and patient preference, phone calls were deemed the optimal telehealth modality.^26^

To guide clinical care, telehealth protocols were designed with cardiology and HF subspecialist input and were modeled on current professional society clinical guidelines.^1,2,3^ The protocols are summarized in eFigure 2 in Supplement 2 and on the trial website.^20^ Protocols were used to identify missing GDMT, assess eligibility for missing therapy, and direct GDMT initiation and titration.^1,2,3^ Eligibility criteria for each therapy is summarized in eTable 1 in Supplement 2.^1,2,3^ Getting patients to take low doses of all 4 therapies (or all eligible therapies) was prioritized by the protocols, with subsequent up-titration.^1^ These protocols were created to facilitate and standardize care as well as to enable future implementation by nonphysician practitioners at sites with limited physician availability.

#### Telehealth Model

Sequential steps and details of the phone-based GDMT optimization telehealth model are summarized in Figure 2. Our telehealth team included a separate team of 2 PCPs (M.M. and D.M.) and a Navajo-speaking nursing assistant (A.T.) to ensure effective communication and incorporate the Navajo Wellness Model.^27^ A cardiologist (L.A.E.) trained the telehealth team to use the protocols and provided ongoing telementoring, with regular virtual check-ins with the team to provide support, review protocols, and perform periodic medical record reviews to ensure implementation was being done per protocol. All medication changes, laboratory work, and home BP and heart rate (HR) readings were documented in the electronic health record (EHR) and flagged the PCP for awareness and to build capacity.

Usual care included routine in-person visits with a clinician. Additionally, all clinicians received a didactic session on updated HFrEF clinical guidelines at the start of the study by a board-certified cardiologist (L.A.E.). With only 1 to 2 Navajo-speaking nurses available for ad hoc interpreting in clinic, family members often assisted with translation.

### Outcomes

The primary outcome was the proportion of patients with an increase in the number of GDMT drug classes filled at 30 days. Patients were only considered to be taking a therapy if the prescription was filled by the patient. Any new class of GDMT was counted. We also counted a transition from an ACEi or ARB to an ARNI as an additional therapy given its superior benefit and stronger clinical recommendation.^1,6^ Any increase in class number was considered a positive outcome and no change or decrease as a negative or null outcome.

The secondary outcomes were an increase in each individual class of GDMT, an increase in dose of currently prescribed GDMT, cardiac referrals made (EHR referral for general cardiology or cardiology subspecialty care), cardiac referrals completed, cardiac procedures or interventions, and HF hospitalizations. Safety outcomes included total adverse events (hyperkalemia [potassium greater than 5.5 mEq/L; to convert to millimoles per liter, multiply by 1], hypokalemia [potassium less than 3.0 mEq/L], HR less than 60 beats per minute, hypotension [systolic BP less than 90 mm Hg], acute kidney injury [creatinine level increase greater than 0.5 mg/dL; to convert to micromoles per liter, multiply by 88.4], volume overload [emergency department visit or emergency clinical encounter for volume overload symptoms], and death). Adverse events were captured through EHR queries at each time point (ie, every 30 days) for all patients. Additionally, we measured PCP comfort with prescribing GDMT for HFrEF (ranging from 1 to 5, with 1 being highly uncomfortable and 5 highly comfortable) through surveys before and after the study.

### Statistical Analysis

Descriptive statistics, including medians and IQRs for continuous variables and counts and frequencies for categorical variables, were presented. The primary end point was the 30-day success rate of addition of a GDMT class. Sample size was estimated using the generalized estimating equation method based on 10 000 simulations. With a sample size of 100, the study provides at least 80% power to detect a 25% clinically significant improvement (ie, the success rate), with a 2-sided type I error of 5%. The assumptions of this power analysis are: (1) the success rates for the control and treatment groups are 10% and 35%, respectively, and (2) the intraclass correlation coefficient is 0.025.

The primary analysis used the intention-to-treat principle. We examined the association between our intervention and outcomes using logistic regression models with generalized estimating equations,^28^ where the first-order autoregressive (AR1) working correlation was used for modeling the intrapatient correlation, determined by the correlation information criterion.^29^ An odds ratio (OR) was used to measure the discrepancy in the proportions of outcomes between intervention and usual care. We reported both unadjusted and adjusted ORs with 95% CIs. Prespecified variables included in regression models (selected a priori as factors known or hypothesized to be associated with GDMT use) were age, sex, left ventricular EF, coronary artery disease, diabetes, and number of GDMT classes at baseline.^15,30^ Statistical significance was determined on the basis of *P* < .05, and all *P* values were 2-tailed. Moreover, we estimated the proportions of outcomes in patients with and without intervention, where the proportions and the 95% CIs were derived from the unadjusted models and the Delta method. For treatment rates of each therapy, only patients eligible for the therapy were included in treatment rate calculations (eTable 1 in Supplement 2).^1^ Given the low occurrence of adverse events, proportions could not be compared for each specific adverse event due to a numerical convergence issue in parameter estimation. Further details on statistical methods can be found in the eMethods in Supplement 2. All analyses were performed using Stata version 15 (StataCorp) and R version 4.3.1 (the R Foundation) with R packages geepack version 1.3.9 and simstudy version 0.7.1.

## Results

Of 103 enrolled American Indian patients, 42 (40.8%) were female, and the median (IQR) age was 65 (53-77) years. The median (IQR) left ventricular EF was 32% (24%-36%). Baseline characteristics by cluster group are summarized in the Table. At baseline, 97 patients (94.2%) were receiving a β-blocker, 90 (87.4%) were receiving an RAAS inhibitor (61 [59.2%], ACEis or ARBs; 29 [28.2%], ARNI), 41 (39.8%) were receiving an MRA, and 45 (43.7%) were receiving an SGLT2i. For detail on the number of baseline therapies by cohort, see eTable 2 in Supplement 2.

**Table.** Baseline Characteristics by Cohort

Characteristic	No. (%)					
	Cohort 1 (n = 21)	Cohort 2 (n = 21)	Cohort 3 (n = 20)	Cohort 4 (n = 20)	Cohort 5 (n = 21)	Total (N = 103)
**Demographic characteristics**						
Age, median (IQR), y	64 (55-68)	64 (58-64)	62 (52-78)	74 (64-80)	62 (49-73)	65 (53-77)
Sex						
Female	9 (43)	7 (33)	9 (45)	8 (40)	9 (43)	42 (41)
Male	12 (57)	14 (67)	11 (55)	12 (60)	12 (57)	61 (59)
American Indian race	21 (100)	21 (100)	20 (100)	20 (100)	21 (100)	103 (100)
**Medical history**						
LVEF, median (IQR), %	28 (22-35)	35 (28-38)	31 (24-36)	28 (22-32)	32 (28-38)	32 (24-36)
Diabetes	13 (62)	14 (67)	12 (60)	9 (45)	15 (71)	63 (61)
Atrial fibrillation	2 (10)	2 (10)	5 (25)	6 (30)	3 (14)	18 (17)
CAD	12 (57)	7 (33)	10 (50)	10 (50)	10 (48)	49 (48)
Hypertension	17 (18)	11 (52)	10 (50)	11 (55)	13 (62)	62 (60)
Dyslipidemia	13 (62)	11 (52)	11 (55)	9 (45)	13 (62)	57 (55)
CKD	6 (29)	5 (24)	8 (40)	7 (35)	9 (43)	35 (34)
Hemodialysis	1 (5)	1 (5)	0	1 (5)	4 (19)	7 (7)
HF etiology						
Ischemic	10 (48)	6 (29)	8 (40)	9 (45)	6 (29)	39 (38)
Nonischemic	10 (48)	10 (48)	9 (45)	7 (35)	11 (52)	47 (46)
Mixed	0	0	0	0	1 (5)	1 (1)
Unknown	1 (5)	5 (24)	3 (15)	4 (20)	3 (14)	16 (16)
HF hospitalization in past 12 mo	9 (43)	10 (48)	9 (45)	7 (35)	14 (67)	49 (48)
**Baseline vital signs**						
Body mass index, median (IQR)^a^	30.0 (26.7-35.4)	29.8 (27.4-33.5)	34.4 (27.2-38.3)	27.1 (24.2-32.8)	26.8 (22.3-31.7)	29.2 (25.3-34.5)
Systolic BP, median (IQR), mm Hg	121 (103-133)	123 (109-133)	127 (114-146)	122 (108-140)	131 (110-145)	123 (108-140)
Diastolic BP, median (IQR), mm Hg	70 (64-82)	74 (70-81)	75 (69-80)	73 (70-82)	73 (67-83)	73 (68-82)
HR, median (IQR), beats per min	78 (71-83)	77 (72-89)	79 (72-92)	70 (65-80)	79 (68-87)	77 (70-87)
**Baseline laboratory findings**						
Potassium, median (IQR), mEq/L	4.3 (3.8-4.5)	4.2 (3.9-4.4)	3.8 (3.6-4.1)	4.1 (3.9-4.4)	4.2 (4.0-4.4)	4.1 (3.8-4.4)
Creatinine, median (IQR), mg/dL	1.00 (0.80-1.20)	1.00 (0.90-1.30)	0.95 (0.70-1.12)	0.95 (0.80-1.75)	1.10 (0.80-2.40)	1.00 (0.80-1.30)
eGFR, median (IQR), mL/min/1.73 m^2^^b^	61 (54-61)	61 (60-61)	61 (60-61)	61 (41-61)	61 (26-61)	61 (51-61)
BNP, median (IQR), pg/mL	226 (34-917)	222 (62-1034)	218 (97-396)	524 (106-2425)	579 (141-896)	275 (76-863)
**Baseline GDMT drug classes**						
β-Blocker^c^						
Entire cohort	19 (91)	21 (100)	18 (90)	19 (95)	20 (95)	97 (94)
Eligible patients, %^d^	91	100	90	100	95	95
ACEi, ARB, or ARNI						
Entire cohort	20 (95)	18 (86)	17 (85)	19 (95)	16 (76)	90 (87)
Eligible patients, %^d^	95	86	100	100	100	96
ACEi						
Entire cohort	7 (33)	7 (33)	6 (30)	8 (40)	8 (38)	36 (35)
Eligible patients, %^d^	33	33	35	47	44	38
ARB						
Entire cohort	5 (24)	7 (33)	4 (20)	4 (20)	5 (24)	25 (24)
Eligible patients, %^d^	24	33	24	24	28	27
ARNI						
Entire cohort	8 (38)	4 (19)	7 (35)	7 (35)	3 (14)	29 (28)
Eligible patients, %^d^	40	22	41	44	21	34
MRA						
Entire cohort	12 (57)	9 (43)	6 (30)	5 (25)	9 (43)	41 (40)
Eligible patients, %^d^	60	53	33	31	50	48
SGLT2i						
Entire cohort	11 (52)	10 (48)	11 (55)	7 (35)	6 (29)	45 (44)
Eligible patients, %^d^	55	50	61	37	35	48
Baseline device therapy						
ICD	2 (10)	0	6 (30)	4 (20)	5 (24)	17 (17)
CRT	2 (10)	2 (10)	2 (10)	3 (15)	0	9 (9)

Abbreviations: ACEi, angiotensin-converting enzyme inhibitor; ARB, angiotensin receptor blockers; ARNI, angiotensin receptor/neprilysin inhibitor; BNP, brain natriuretic peptide; BP, blood pressure; CAD, coronary artery disease; CKD, chronic kidney disease; CRT, cardiac resynchronization therapy; eGFR, estimated glomerular filtration rate; GDMT, guideline-directed medical therapy; HF, heart failure; HR, heart rate; ICD, implantable cardioverter-defibrillator; LVEF, left ventricular ejection fraction; MRA, mineralocorticoid receptor antagonist; SGLT2i, sodium-glucose cotransporter 2 inhibitor.

SI conversion factor: To convert potassium to mmol/L, multiply by 1; creatinine to µmol/L, multiply by 88.4; BNP to ng/L, multiply by 1.

^a^ Calculated as weight in kilograms divided by height in meters squared.

^b^ The Indian Health Service laboratory reports any eGFR greater than 60 mL/min/1.73 m^2^ as “>60” without a discrete number. Therefore, for analyses, 61 was used as the eGFR in these cases.

^c^ β-Blockers included evidence-based β-blockers metoprolol succinate and carvedilol, except 1 patient in cohort 5 who was taking labetalol.

^d^ Eligible patients determined using contraindications, allergies, vital sign, and laboratory value cutoffs from the American College of Cardiology/American Heart Association Guideline for Management of Heart Failure and the American College of Cardiology Expert Consensus Pathway for Optimization of Heart Failure Treatment (eTable 1 in Supplement 2).

### Outcomes

**Figure 3.**
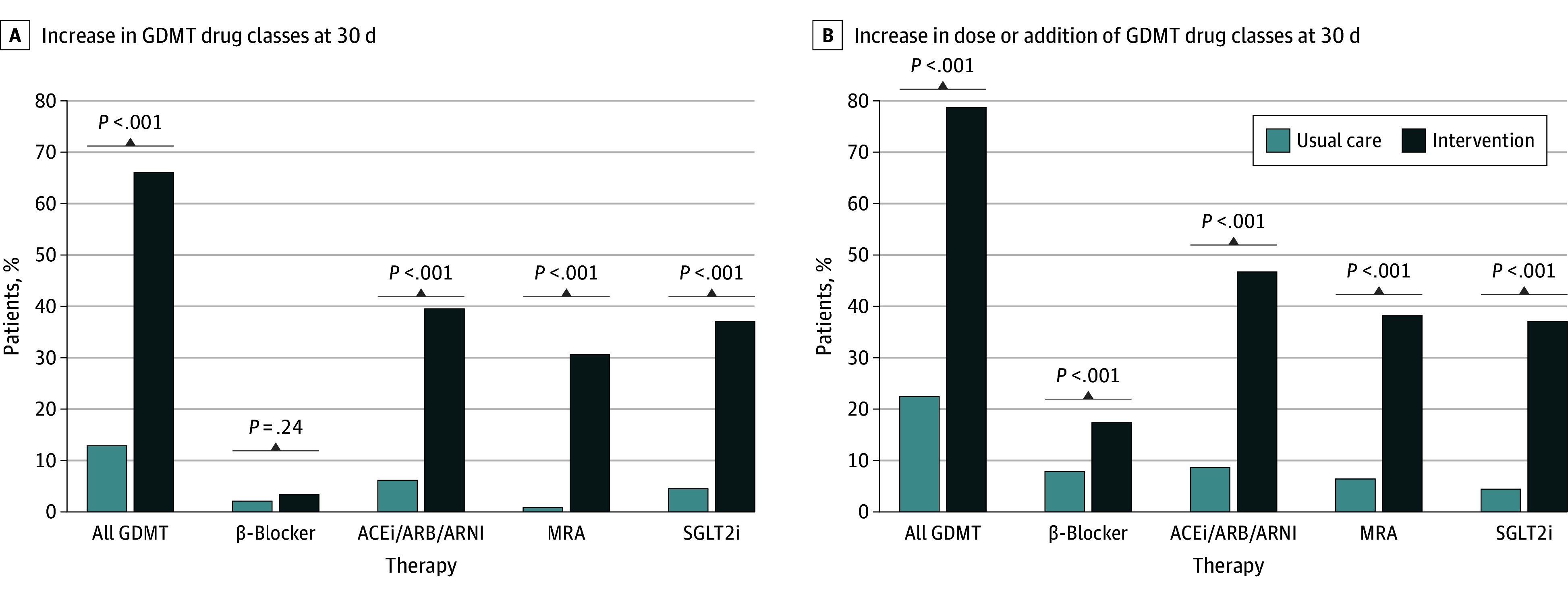
Primary and Secondary Outcomes According to Intervention (Telehealth) vs Usual Care A, The primary outcome was the proportion of patients with an increase in the number of guideline-directed medical therapy (GDMT) drug classes filled at 30 days. B, The secondary outcome was the proportion of patients with an increase in the number of GDMT drug classes or dose of drug at 30 days. As demonstrated, there was a significant improvement in the number and doses of GDMT in the intervention arm compared with the usual care arm. For the primary outcome, any new class of GDMT was counted. In addition, a transition from an angiotensin-converting enzyme inhibitor (ACEi) or angiotensin receptor blocker (ARB) to an angiotensin receptor–neprilysin inhibitor (ARNI) was considered as an additional therapy given its superior benefit and stronger clinical recommendation (therefore, ACEi/ARB/ARNI rates also include patients that switched from ACEi/ARB to ARNI therapy). MRA indicates mineralocorticoid receptor antagonist; SGLT2i, sodium-glucose cotransporter-2 inhibitor.

**Figure 4.**
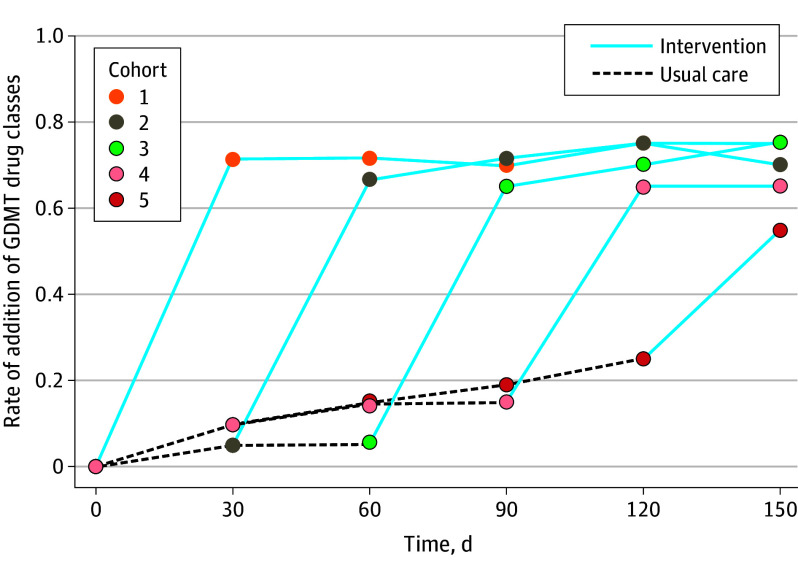
Rates of the Primary Outcome Over Time According to Cluster (Intervention Time) vs Usual Care Cluster 1 (n = 21) had immediate implementation of the telehealth model, while cluster 2 (n = 21) received the intervention at 30 days, cluster 3 (n = 30) at 60 days, cluster 4 (n = 20) at 90 days, and cluster 5 (n = 21) at 120 days. The solid line represents the time when a cohort was in the intervention arm, and the dashed line represents when the cohort was in the usual care arm. As demonstrated, there was a significant and rapid increase in the number of guideline-directed medical therapy (GDMT) drug classes and rates of the primary outcome as clusters crossed over into the intervention arm.

The primary outcome was observed in 66.2% of patients in the intervention arm and 13.1% in the usual care arm (unadjusted OR, 12.99; 95% CI, 6.87-24.53; *P* < .001; adjusted OR, 26.39; 95% CI, 10.20-68.28; *P* < .001) (eTable 3 in Supplement 2). While there were increases in prescription of each GDMT drug class in both study arms, there were more significant increases in the intervention arm compared with usual care, except for β-blockers (Figure 3). The number of patients needed to receive the intervention to result in the addition of a GDMT drug class was 1.88. Rates of the primary outcome by cluster over time are shown in Figure 4. At the end of the study, 96 of 99 eligible patients (97%) were taking a β-blocker, 89 of 91 (98%) were taking an RAAS inhibitor (ie, ACEi, ARB, or ARNI); 60 of 77 (78%) were taking an ARNI, 65 of 77 (84%) were taking an SGLT2i, and 60 of 77 (78%) were taking an MRA, with 58 of 72 patients (81%) eligible for all 4 medications receiving quadruple therapy.

The secondary outcome of an increase in dose or addition of a class of GDMT was observed in 79.0% in the intervention arm and 22.6% in the usual care arm (unadjusted OR, 12.90; 95% CI, 7.24-22.98; adjusted OR, 18.00; 95% CI, 8.85-36.60) (Figure 3; eTable 4 in Supplement 2). Spaghetti plots of the secondary outcome (addition or increase in dose of GDMT class) and the addition of each individual therapy and dose increases by cohort over time are shown in eFigures 3 to 10 in Supplement 2. GDMT rates at 5 months for cohort 1 and at 3 months for cohorts 2 and 3 are shown in eTable 5 in Supplement 2.

There were no statistically significant differences in the rates of cardiology referrals (21.7% vs 16.8%; *P* = .16), completed referrals (11.0% vs 10.8%; *P* = .95), or cardiac interventions (5.0% vs 2.3%; *P* = .15) between the intervention and usual care arms (eTable 6 in Supplement 2). There were fewer HF hospitalizations in the intervention arm (1.3% vs 4.3%; OR, 0.30; 95% CI, 0.11-0.85; *P* = .02). There was 1 death in the usual care arm. There were few adverse events and no significant differences in total adverse events between the intervention and usual care arms (6.6% vs 5.0%; *P* = .51) (eTable 7 in Supplement 2). PCP comfort with prescribing GDMT (range, 1 to 5) increased from a baseline mean (SD) of 1.86 (0.86) to 4.36 (0.63) at conclusion of the study (*P* < .001).

Of planned telehealth phone visits, 83 of 103 (80.5%) were conducted successfully. Of these, 59 (71%) completed home BP and HR measurements, and 55 (66%) completed laboratory work per protocol.

## Discussion

In this HF trial in rural Navajo Nation using a phone-based HFrEF optimization model in a rural setting—to our knowledge, the first of its kind—the Hózhó randomized clinical trial demonstrates that phone-based GDMT optimization with remote telemonitoring led to improved rates of GDMT at 30 days compared with usual care. There were fewer HF hospitalizations and no differences in adverse events among those in the intervention vs usual care arm.

Other EHR-based strategies have been shown to be modestly effective in increasing rates of GDMT and often rely on in-person visits with a clinician.^13,14,15,16^ The most marginalized patient groups often face barriers to accessing care, especially cardiology care.^31,32,33,34^ Even when seen by a clinician, racially marginalized groups have lower rates of prescription for guideline-recommended therapies, including for HFrEF.^35,36,37,38^ Our strategy leveraged the EHR on a health system level to identify patients not receiving appropriate therapy and subsequently optimize therapy without relying on in-person visits for specialty care; such models advance equity and combat structural racism.^39,40,41,42,43^

Our trial demonstrates that effective strategies to improve GDMT rates must be community-designed and tailored to fit the local context.^25^ This strategy was designed with community stakeholder input to meet community needs and address unique Indigenous determinants of health.^44^ Cardiovascular health disparities among American Indian and Alaska Native people are due to the enduring impacts of settler colonialism^45,46,47,48,49^; 1 in 3 people living on the Navajo Reservation lack running water or electricity, and significant access issues include lack of paved roads and transportation.^19,50,51,52^ IHS sites are chronically underfunded by the US government, and specialty access is limited.^53,54^ By centering communities to design programs to prioritize care delivery for racially marginalized groups, equity in HF care can be achieved.^55^

Our team included a Diné (Navajo)-speaking nursing assistant (A.T.) who contacted patients to discuss recommendations and to align Western medicine with Diné health frameworks. While the study was not designed to evaluate the relative contribution of different components of the model, we strongly believe that offering culturally and linguistically competent care contributed to high rates of patient uptake of recommendations and is particularly important when designing telehealth models aimed at marginalized patient groups.

Telehealth has been shown to be an effective way to reach patients in rural settings, including for cardiology care.^56^ This model differs from traditional telehealth care in that it is a targeted optimization strategy, which fully offloads the burden from PCPs, does not rely on clinician or specialty availability or scheduled telehealth visits (ie, calls can occur at any time), encompasses a health system–level strategy to identify patients who are not receiving optimized care, and allows for rapid optimization of therapy.

This model led to rapid uptake of GDMT therapy within 30 days, which is critically important given the early benefit of these therapies to improve mortality and lower HF hospitalizations.^6,57,58,59,60^ With this model, our rates of GDMT far exceeded national rates and results from other intervention studies.^9,15,16^ This study also provides further evidence of the safety of remote initiation and titration strategies for GDMT.^61^ In addition, our baseline data on GDMT rates provides the first evaluation, to our knowledge, of the quality of HFrEF care in an American Indian and IHS cohort in the current era.

Although this was a secondary outcome, we found lower rates of HF hospitalizations with our model. In addition to the known benefits of GDMT,^4,5,6,7,8^ this could also be due to early identification and rectification of issues, such as running out of medications, or early signs of volume overload. This should be explored further in subsequent studies.

### Limitations

This study has several limitations. This study may not be generalizable due to the small sample size and setting. Results may not be generalizable to other IHS sites or other health systems. Medications are provided free of cost to enrolled IHS patients. This model may not be as successful in other payer settings, especially for uptake of newer medications, such as SGLT2i. We evaluated filled prescriptions but did not evaluate adherence after prescription pick-up. Not all patients in the usual care arm were able to have an in-person clinical visit within 30 days or even during the study period. However, as a pragmatic trial, this reflects real-world conditions in which clinician availability and transportation are limited. This model was designed with stakeholder input to optimize acceptability and center local priorities. Expansion to other settings would require similar tailoring to fit the local context, with additional program evaluation. We are currently expanding to another large IHS site in Arizona; ongoing evaluations will help better understand issues of scalability and generalizability. This intervention was tested after a cardiologist (L.A.E.) provided a lecture to all clinicians on updated clinical guidelines and recommendations for GDMT use. Additionally, PCPs were flagged on all medication changes to help build capacity, which is reflected in GDMT increases in the nonintervention arm (with greater increases over time). Carry-over effects are an inherent limitation to the closed-cohort design of a stepped-wedge trial.^22^ However, this potential for contamination would tend to bias results toward the null. Adverse events are likely underestimated given not all patients performed BP and HR measurements and laboratory work per protocol. Given the small number of adverse events and smaller sample size, we could not detect significant differences in adverse events. Monitoring of adverse events will be critical as this model is expanded locally and to other IHS sites. Additionally, the follow-up time in this study was short. Evaluating longer-term adherence and outcomes is the subject of future work and will be important to characterize the durability of the intervention effects.

## Conclusions

A telehealth model leveraging phone-based GDMT optimization with remote telemonitoring led to significant and rapid increases in the uptake of GDMT for HFrEF. This low-cost strategy could be expanded to other rural settings where access to care is limited.

## Notes

Supplement 1. Trial Protocol

Supplement 2. **eFigure 1.** Map of Trial Sites and Setting **eFigure 2.** Flowcharts/Protocols for Telehealth Model **eTable 1.** Eligibility/Exclusion Criteria for Each GDMT Therapy **eTable 2.** Number of Guideline-Directed Medical Therapies by Cohort at Baseline **eTable 3.** Multivariable Analyses of the Association of Intervention (Telehealth Model) With the Primary Outcome (Addition of GDMT Class at 30 Days) **eTable 4.** Multivariable Analyses of the Association of Intervention (Telehealth Model) With the Secondary Outcome of Addition of or Increase in Dose of GDMT **eFigure 3.** Spaghetti Plot of Secondary Outcome (Addition of GDMT Drug Class or Increase in GDMT Drug Dose) Over Time by Cohort **eFigure 4.** Spaghetti Plot of ACEi/ARB/ARNI Addition Over Time by Cohort **eFigure 5.** Spaghetti Plot of β-Blocker Addition Over Time by Cohort **eFigure 6.** Spaghetti Plot of MRA Addition Over Time by Cohort **eFigure 7.** Spaghetti Plot of SGLT2i Addition Over Time by Cohort **eFigure 8.** Spaghetti Plot of Addition or Increase in Dose of ACEi/ARB/ARNI Over Time by Cohort **eFigure 9.** Spaghetti Plot of Addition or Increase in Dose of β-Blocker Over Time by Cohort **eFigure 10.** Spaghetti Plot of Addition or Increase in Dose of MRA Over Time by Cohort **eTable 5.** Longer-Term GDMT Rates for Cohorts 1-3 **eTable 6.** Cardiac Procedures/Interventions by Study Arm **eTable 7.** Adverse Events Over Time for the Cohort **eMethods.** Additional Statistical Analysis Details on Power Analysis, Delta Method Used to Derive Confidence Intervals, and Success Rates

Supplement 3. Data Sharing Statement
