# Supplementary material for: Telephone-Based Guideline-Directed Medical Therapy Optimization in Navajo Nation: The Hózhó Randomized Clinical Trial
Source: JAMA Intern Med. 2024 Apr 7;184(6):681–90. doi: 10.1001/jamainternmed.2024.1523 (PMC11000136; doi:10.1001/jamainternmed.2024.1523)
Supplement: Supplement 1. — Trial Protocol [file jamainternmed-e241523-s001.pdf]

PROTOCOL TITLE: Hózhó (Heart Failure **O**ptimi**Z**ation at **H**ome to Improve **O**utcomes) Quality Improvement Model for HF Patients Receiving Care through the Indian Health Service in Navajo Nation

Version Date 9/10/2022

Clinicaltrials.gov Registration # NCT05792085

SECTION 1: RESEARCH PLAN

Statement of Purpose:

Heart failure is the leading cause of hospitalization among older adults and has a 5-year mortality of up to 50%.[1][2]. There are well-established evidence-based guidelines for treating heart failure with reduced ejection fraction (HFrEF), a major subset of HF. The background of guideline-directed medical therapy (GDMT) for HFrEF now includes 4 classes of therapy: a beta-blocker, an angiotensin-converting enzyme inhibitor (ACE) inhibitor/angiotensin receptor blocker (ARB) or preferably an angiotensin receptor-neprilysin inhibitor (ARNI), a mineralocorticoid receptor antagonist (MRA), and a sodium glucose transporter 2 (SGLT2) inhibitor [3-7]. There is a cumulative risk reduction for mortality of >75% among HF patients receiving combined therapy with a beta-blocker, ARNI, MRA and SGLT2 inhibitor.[3] Therefore, the American College of Cardiology/American Heart Association guidelines recommend treatment with all 4 of these agents and titration to highest-tolerated dose (or target dose) to maximize clinical benefit.[8] However, despite clear evidence-based interventions for HF, suboptimal care is a major driver of poor HF-related outcomes in the U.S.[9] Guideline-directed therapies for HFrEF are underutilized among all patients in the U.S.[10]. AI/AN patients receiving care through the Indian Health Service, in particular, are at high risk of not receiving appropriate GDMT given barriers to accessing appropriate care.[11]

The reasons for underutilization of appropriate GDMT in HF are multifactorial. In the Indian Health Service, access to cardiology care is severely limited, and HF care is provided primarily by primary care providers. We surveyed primary providers at two Indian Health Service sites to identify the primary barriers to getting HFrEF patients on appropriate GDMT. The primary barriers identified were include lack of knowledge/comfort among providers about guidelines and appropriate management, clinical burden, time constraints during the visit. Given this we hypothesized that a model that identified HFrEF patients not on appropriate therapy, and initiated missing recommended therapy by the study team would be an effective way to improve HF quality of care and uptake of GDMT. In addition, given the rurality of the Navajo patients cared for at these two IHS sites, and the barriers to accessing care, we

hypothesized that a telemonitoring model in which patients had GDMT initiation and uptitration at home, with home BP monitoring would be preferable.

We therefore, in discussion with community members and primary providers at two IHS sites in Navajo, designed a model to identify HFrEF patients cared for in the system, identify gaps in their therapy, and initiate appropriate therapy by the study team with home BP and HR monitoring for initiation and titration of GDMT. We propose a stepped wedge randomized trial to compare the implementation of this model compared to usual care over a 6-month period with 5, 30-day cross over periods. The primary outcome for the trial will be the proportion of patients who have an increase in the number of prescribed evidence-based therapies for HFrEF (betablockers, ACE-I/ARB/ARNI, MRA, SGLT2i) 30 days post implementation. This endpoint is based on filling of the prescription. In addition to addition of missing GDMT, we will also consider a change from an ACEI/ARB to an ARNI consistent with increase in the 'number' of GDMT given their demonstrated benefit. Secondary outcomes will include: percentage of patients prescribed each medication class (i.e. percentage on beta-blockers, percentage on ACE-I/ARB, percentage of ARNIs, percentage on SGLT2i, and percentage on MRAs), medication doses, all at 30-days as well as post-implementation at 6 months; HF hospitalizations at each time point, 6-month mortality, adverse events (to include hypokalemia [K<3.0 mEq/L], hyperkalemia [K >5.5 mEq/L], AKI [defined as Cr increase >0.3 from baseline], Hyponatremia [Na<130 mg/dl], volume overload [urgent clinic visit/ ER visits for lower extremity edema, dyspnea, with clinical evaluation consistent with volume overload]. Outcomes determined by review of medical, hospital, and billing records.

Probable Duration of Project: 6 months

Research Plan: We plan to conduct a randomized, stepped-wedge design trial, with 5 clusters, with 5 crossover points at every 30 days, for a total of 6 months. This will be done at two IHS clinical sites in the Gallup Service Unit at Indian Health Service: Gallup Indian Medical Center and Tohatchi Health Clinic.

Study Population: All patients with ICD code I50* (given ICDI50.2 may not always be selected) and echo with LVEF <=40% within the last 24 months (24 months was chosen rather than 12 months given limited echo availability, particularly during COVID-19). Only patients with active prescription through IHS in the last 12 months and engaged in care at our centers (clinical visit within last 12 months, with primary care physician at one of the two sites) will be included. Patients will be randomized to 1 of 5 clusters (which determines time of implementation).

Our study design is as follows:

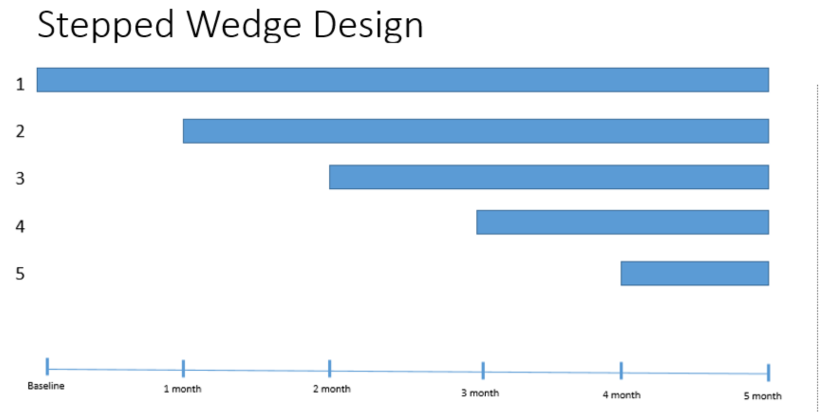

Subject Recruitment: Eligible patients are identified as above. All primary providers will be sent a message through the EHR in the patient chart reporting that their patient is planned to be enrolled in the study. Providers can sign to consent to patient enrollment, or otherwise, can opt out of the study if their feel patient enrollment not appropriate.

Eligible patients are identified through a query through iCARE, a EHR based data system through the IHS EHR. Patients with ICD10 diagnostic criteria for HF will be identified, and then all those patients will have their chart reviewed to confirm LVEF less than or equal to 40% on echo within last 24 months. The inclusion criteria will be all adults ≥18 years, who have a prescription in the IHS system in the last 12 months and have had a clinical visit at GIMC or THC in the last 12 months. As this is an intervention assessing a model to improve uptake of standard of care, we cannot inform patients of their participation in the study at the time of enrollment, as this would contaminate the randomized exposure. As this study presents minimal risk to patients, we requested a waiver of informed consent at the patient level.

Randomization

Randomization in this study will occur at the level of the patient. This is a stepped wedge trial design, and patients will be randomized to 1 of 5 clusters. Every cluster will have the model implemented, but the time point of implementation will vary based on cluster, in 30 day increments. Cluster 1 will have the model implemented at time 0, cluster 2 at 30 days, cluster 3 at 60 days, etc. Prior to implementation, patients in pre-implementation time points will receive usual care. In addition, all providers received a lecture regarding updated ACC/AHA/HFSA guidelines and expert consensus prior to implementation of the model. Given the stepped-wedge design, all patients will eventual benefit from implementation of the model, but it also allows us to evaluate the effect of secular trends towards better care over time (and ensure the model rather than trends in care are not responsible for improving care).

Intervention

This project was implemented as an Indian Health Service Innovations Award to improve quality of care for patients. Once patients are at their intervention time point, they receive implementation of our designed HF QI model. As part of this model, all patients are given a home BP cuff (Omron 5 arm cuff) for home BP monitoring. Protocols for each step of this model are detailed in the Supplemental materials. However, in brief, as part of the intervention, these protocols allow the study team to identify which, if any recommended guideline-directed mediations are missing, identify any contraindications to therapy, and initiate therapy as directed if no contraindications are identified. Prescriptions for the therapy are made, appropriate follow-up lab testing ordered if needed, and patients are called by the study team to discuss the therapy and recommendations. Getting patients are low doses of all 4 therapy (or all therapies they are eligible on) is prioritized by the study protocol, and then doses could be secondarily uptitrated per recent HF Expert Consensus Statement.[8] Utilizing home blood pressure monitoring, medications were added and titrated as directed by study protocols, with patients coming in for lab work as recommended by the study protocol. All medication changes, lab work, home BP and HR readings, will be documented in the EHR and sent to the PCP. Please see supplement for flowcharts utilized in this study.

<u>Endpoints:</u> The primary outcome for the trial will be the proportion of patients who have an increase in the number of prescribed evidence-based therapies for HFrEF (betablockers, ACE-I/ARB/ARNI, MRA,

SGLT2i) 30 days post implementation. This endpoint is based on filling of the prescription. In addition to addition of missing GDMT, we will also consider a change from an ACEI/ARB to an ARNI consistent with increase in the 'number' of GDMT given their demonstrated superior benefit.

Secondary outcomes will include: percentage of patients prescribed each medication class (i.e. percentage on beta-blockers, percentage on ACE-I/ARB, percentage of ARNIs, percentage on SGLT2i, and percentage on MRAs), medication doses, all at 30-days as well as post-implementation at 6 months; HF hospitalizations, 6-month mortality, adverse events (to include hypokalemia [K<3.0 mEq/L], hyperkalemia [K >5.5 mEq/L], AKI [defined as Cr increase >0.3 from baseline], Hyponatremia [Na<130 mg/dl], volume overload [urgent clinic visit/ ER visits for lower extremity edema, dyspnea, with clinical evaluation consistent with volume overload]. Outcomes determined by review of medical, hospital, and billing records.

<u>Blinding of Intervention:</u> Patients will be informed that they are enrolled in a new quality improvement program to get them on optimal therapy and thus will not be blinded to their randomization status or participation in this trial. Provider subjects will, obviously, not be blinded to the intervention as they are receiving the alert and will be consenting to participate in the study. We will engage in both pre-trial and periodic teaching and discussion with all participating care providers to inform clinicians about the nature of the study and to discuss specific factors that are being measured. The study team is not blinded to the cluster assignment as they are implementing the model. However, the outcomes will be pulled from the EHR from study members, and those performing the analysis are blinded to randomization/cluster status of the patient.

<u>Intervention Duration</u>: The study team will implement the model starting when a patient cluster's planned implementation start until the end of the study period (6 month total).

Genetic Testing: N/A no biological material will be collected on patients.

1. Subject Population: For ethical considerations, we aim to enroll all patients who meet eligibility criteria at GIMC and THC. Subjects who will be enrolled are those with a diagnosis of HFrEF (LVEF ≤ 40%) who are seen in an outpatient internal medicine or family medicine IHS clinics at GIMC or THC
2. Subject Classification: N/A

Inclusion/Exclusion Criteria

Inclusion

- Age >=18 years
- Seen at internal medicine or family medicine clinic at GIMC or THC in last 12 months with primary care physician at one of the two sites
- Left ventricular ejection fraction ≤40%
- Prescription in IHS system in past 12 months

Exclusion

- Primary providers opted out of inclusion
- Hospice care
- Living in acute rehabilitation or skilled nursing facility

How will eligibility be determined, and by whom?

Eligibility of patients will be assessed by the study team based on the patient's medical record. However, as detailed, if provider feels patient is inappropriate for enrollment, they can also opt out of enrollment. Those who meet criteria will have a EHR message sent through the chart to the primary provider. Primary providers have 1 week to opt out. If they do not opt out, patient will be automatically enrolled and placed into a randomization cluster group.

Protection of Human Subjects: Protection of Human Subjects:

This study is aimed at implementing a model to get patients on appropriate standard of care, guideline directed therapy. Therefore, this poses a minimal risk to patients. We have met with HF experts at the University of Pennsylvania, primary care providers, QI and safety officers at the IHS, nurses to ensure that the design of this study, including the implemented protocols, minimize any risk to the patients. We will restrict the study only to GIMC and THC. Our Data Safety Monitoring committee will closely monitor for any significant adverse events that may require early discontinuation of the trial.

Human subjects' involvement, characteristics and design: The studies outlined in this proposal depend on the enrollment of individuals with heart failure. No vulnerable populations are being specifically targeted. We are limiting enrollment to individuals above age 18 years as the etiology and practices surrounding heart failure in pediatrics populations differ significantly from those in adults. All data is transmitted in encrypted and secure fashion, stored on servers with "triple-lock" certification, and is available only to members of the study team, IRB, and any state or federal agencies with auditing power.

Sources of Materials: No biological materials will be obtained or stored as part of these studies. Only data, as collected during set time points from the EHR will be obtained. Data includes medical record elements such as demographics, pharmacy records such as medication prescription and dosing, laboratory values, and administrative codes. All data will be stored without PHI. However, we will retain a linking dataset to be able to re-link individual data to actual patients for future studies and ongoing efforts through the HIS. Access to individually identifiable information will be limited to the PI of the study, and only then via a linking file as aforementioned. All data used for analysis and dissemination to other investigators will be de-identified.

Over or under treatment: Implementation of the model, and all of the medication changes will be sent to the primary provider. It is possible that not only those patients, but then other patients cared for by those providers may be more likely to be started on evidence based medical therapies. These interventions fall within the standard-of-care and may benefit patients, but it is also possible that additional interventions may not benefit patients and could incur additional costs. However, this is what we are testing as part of this pragmatic trial.

Potential benefits of the proposed research to the subjects and others: Subjects in this study may directly benefit from being started on evidence-based therapies for their heart failure. In addition, providers will be able to see implementation and medication changes, which may improve their

education and comfort with updated HFrEF guidelines, leading to improved care for other patients. Additionally, regardless of the outcome for participants, the results of these studies may lead to significant benefit in the IHS and other health systems where access to care, especially cardiology care is limited. This model could be similarly expanded to other sites and to other disease entities. The risk/benefit ratio, given the minimal risk to study subjects, is more than acceptable in this series of studies.

Data and Safety Monitoring Plan:

• What is the investigator's assessment of the overall risk level for subjects participating in this study?

We believe that this poses minimal risk to the patients.

• If children are involved, what is the investigator's assessment of the overall risk level for the children participating in this study? No children will be involved.

This study poses **i. Minimal risk** ii. Greater than minimal

The principal investigators (PI) is responsible for monitoring the data, assuring protocol compliance, and conducting the safety reviews at the specified frequency regularly. During the review process the PIs will evaluate whether the study should continue unchanged, require modification/amendment, or close to enrollment. The PIs or the Nation Nation Human Research Review Board (Navajo Nation IRB) have the authority to stop or suspend the study or require modifications.

This protocol presents minimal risks to the subjects and Unanticipated Problems Involving Risks to Subjects or Others (UPIRSOs), including adverse events, are not anticipated. In the unlikely event that such events occur, Reportable Events (which are events that are serious or life-threatening and unanticipated (or anticipated but occurring with a greater frequency than expected) and possibly, probably, or definitely related) or Unanticipated Problems Involving Risks to Subjects or Others that may require a temporary or permanent interruption of study activities will be reported immediately (if possible), followed by a written report within 5 calendar days of the Principal Investigator becoming aware of the event to the IRB (using the appropriate forms from the website) and any appropriate funding and regulatory agencies. The investigator will apprise fellow investigators and study personnel of all UPIRSOs and adverse events that occur during the conduct of this research project through regular study meetings and via email as they are reviewed by the principal investigator.

Statistical Considerations

Power analysis: The primary endpoint of this revised stepped wedge randomized trial (SW-CRT) design, with 5 time periods, is the 30-day success rate. The sample size estimation was completed using the generalized estimating equations (GEE) method based on 10,000 simulations. With the proposed sample size of 100, the study provides at least 80% power to detect a 25% clinically significant improvement in the success rate, with a two-sided type I error of 5%. The assumptions of this power analysis are as follows: (1) the success rates for the control and treatment groups are 10% and 35%, respectively, and (2) the intraclass correlation coefficient (ICC) is 0.025.

However, given ethical concerns, we will plan to enroll all patients that meet eligibility criteria at the two IHS sites, and given the stepped-wedge design of the trial, all patients will receive the intervention, just at varying time points.

Interim Analysis: We will not perform interim safety or efficacy analyses given short trial duration, but the DSMB will closely monitor for any unexpected adverse events. Earlier stopping will be considered for the reason of safety by the DSMB if any significant adverse events. This is a novel intervention, and we are obligated to ensure the safety of this model and that it is minimal risk.

Statistical Analysis:

The primary analysis used the intention-to-treat principle: we examined the association between our intervention and outcomes using logistic regression models with the generalized estimating equations, where the AR1 working correlation will be employed for modeling the intra-patient correlation, determined by the correlation information criterion. We will report both unadjusted and adjusted logOR as well as the 95% confidence intervals. The variables used for adjustment in regression models were prespecified, and included the baseline characteristics: age, sex, LVEF, coronary artery disease, disease, diabetes, and number of GDMT classes at baseline. Statistical significance was determined on the basis of $P < 0.05$. Moreover, we also estimated the proportions of outcomes in patients with and without intervention, where the proportions and the CIs were derived from the unadjusted models and the Delta method.

Assessment of contamination: Providers may learn to better treat heart failure as consistent with evidence-based therapies over time. Given we are not clustering by provider, but rather patients, it is possible that providers will take what is being implemented on their patient that is in an active implementation phase and apply it to a patient in a non-active implementation phase. Therefore, there is a high risk of contamination. However, with the stepped wedge design, we will be able to also see how GDMT rates increase over the study period for those clusters who are not immediately in an active implementation arm. We will be able to better understand if increases in GDMT are related to the intervention or just due to increases over time due to improved knowledge of providers over time. However, this potential for contamination would bias our results towards the null.

References

1. Jencks SF, Williams MV, Coleman EA. Rehospitalizations among patients in the Medicare fee-for-service program. N Engl J Med. 2009 Apr 2;360(14):1418-28. doi: 10.1056/NEJMsa0803563. Erratum in: N Engl J Med. 2011 Apr 21;364(16):1582. PMID: 19339721.
2. Levy D, Kenchaiah S, Larson MG, Benjamin EJ, Kupka MJ, Ho KK, Murabito JM, Vasan RS. Long-term trends in the incidence of and survival with heart failure.N Engl J Med. 2002; 347:1397–1402. doi: 10.1056/NEJMoa020265. PMCID: PMC2729712.
3. Fonarow GC, Yancy CW, Hernandez AF, Peterson ED, Spertus JA, Heidenreich PA. Potential impact of optimal implementation of evidence-based heart failure therapies on mortality. Am Heart J. 2011 Jun;161(6):1024-30.e3. doi: 10.1016/j.ahj.2011.01.027. PMID: 21641346.
4. Yancy CW, Jessup M, Bozkurt B, Butler J, Casey DE Jr, Drazner MH, Fonarow GC, Geraci SA, Horwich T, Januzzi JL, Johnson MR, Kasper EK, Levy WC, Masoudi FA, McBride PE, McMurray JJ, Mitchell JE, Peterson PN, Riegel B, Sam F, Stevenson LW, Tang WH, Tsai EJ, Wilkoff BL; American College of Cardiology Foundation; American Heart Association Task Force on Practice Guidelines. 2013 ACCF/AHA guideline for the management of heart failure: a report of the American College of Cardiology Foundation/American Heart Association Task Force on Practice Guidelines. J Am Coll Cardiol. 2013 Oct 15;62(16):e147-239. doi: 10.1016/j.jacc.2013.05.019. Epub 2013 Jun 5. PMID: 23747642.
5. McMurray JJ, Packer M, Desai AS, Gong J, Lefkowitz MP, Rizkala AR, Rouleau JL, Shi VC, Solomon SD, Swedberg K, Zile MR; PARADIGM-HF Investigators and Committees. Angiotensin-neprilysin inhibition versus enalapril in heart failure. N Engl J Med. 2014 Sep 11;371(11):993-1004. doi: 10.1056/NEJMoa1409077. Epub 2014 Aug 30. PMID: 25176015.
6. McMurray JJV, Solomon SD, Inzucchi SE, Køber L, Kosiborod MN, Martinez FA, Ponikowski P, Sabatine MS, Anand IS, Bělohlávek J, Böhm M, Chiang CE, Chopra VK, de Boer RA, Desai AS, Diez M, Drozdz J, Dukát A, Ge J, Howlett JG, Katova T, Kitakaze M, Ljungman CEA, Merkely B, Nicolau JC, O'Meara E, Petrie MC, Vinh PN, Schou M, Tereshchenko S, Verma S, Held C, DeMets DL, Docherty KF, Jhund PS, Bengtsson O, Sjöstrand M, Langkilde AM; DAPA-HF Trial Committees and Investigators. Dapagliflozin in Patients with Heart Failure and Reduced Ejection Fraction. N Engl J Med. 2019 Nov 21;381(21):1995-2008. doi: 10.1056/NEJMoa1911303. Epub 2019 Sep 19. PMID: 31535829.
7. Packer M, Anker SD, Butler J, Filippatos G, Pocock SJ, Carson P, Januzzi J, Verma S, Tsutsui H, Brueckmann M, Jamal W, Kimura K, Schnee J, Zeller C, Cotton D, Bocchi E, Böhm M, Choi DJ, Chopra V, Chuquiure E, Giannetti N, Janssens S, Zhang J, Gonzalez Juanatey JR, Kaul S, Brunner-La Rocca HP, Merkely B, Nicholls SJ, Perrone S, Pina I, Ponikowski P, Sattar N, Senni M, Seronde MF, Spinar J, Squire I, Taddei S, Wanner C, Zannad F; EMPEROR-Reduced Trial Investigators. Cardiovascular and Renal Outcomes with Empagliflozin in Heart Failure. N Engl J Med. 2020 Oct 8;383(15):1413-1424. doi: 10.1056/NEJMoa2022190. Epub 2020 Aug 28. PMID: 32865377.
8. 2021 Update to the 2017 ACC Expert Consensus Decision Pathway for Optimization of Heart Failure Treatment: Answers to 10 Pivotal Issues About Heart Failure With Reduced Ejection Fraction: A Report of the American College of Cardiology Solution Set Oversight Committee. J Am Coll Cardiol 2021;Jan 11:[Epub ahead of print].
9. Michalsen A, König G, Thimme W. Preventable causative factors leading to hospital admission with decompensated heart failure. Heart. 1998 Nov;80(5):437-41. doi: 10.1136/hrt.80.5.437. PMID: 9930040; PMCID: PMC1728853.
10. Greene SJ, Butler J, Albert NM, DeVore AD, Sharma PP, Duffy CI, Hill CL, McCague K, Mi X, Patterson JH, Spertus JA, Thomas L, Williams FB, Hernandez AF, Fonarow GC. Medical Therapy

for Heart Failure With Reduced Ejection Fraction: The CHAMP-HF Registry. J Am Coll Cardiol. 2018 Jul 24;72(4):351-366. doi: 10.1016/j.jacc.2018.04.070. PMID: 30025570.
11. Hutchinson RN, Shin S. Systematic review of health disparities for cardiovascular diseases and associated factors among American Indian and Alaska Native populations. PLoS One. 2014 Jan 15;9(1):e80973. doi: 10.1371/journal.pone.0080973. PMID: 24454685; PMCID: PMC3893081.

Tables

Table 1. Baseline Demographic and Clinical Characteristics of the Cohort

Characteristic	Value
Age, median [IQR]	
Sex, n (%)	
Race, n (%)	
American Indian	
BMI, kg/m2, median [IQR]	
Comorbidities	
Diabetes, n (%)	
Atrial fibrillation, n (%)	
CAD, n (%)	
Hypertension, n (%)	
Hyperlipidemia, n (%)	
CKD, n (%)	
Stage I, n (%)	
Stage II, n (%)	
Stage III, n (%)	
Stage IV, n (%)	
Stage V/ESRD, n (%)	
On HD, n (%)	
HF etiology, n (%)	
Ischemic, n (%)	
Nonischemic, n (%)	
Unknown, n (%)	
Prior Stress Test, n (%)	
Prior Coronary Angiogram, n (%)	
Prior Cardiac MRI, n (%)	
Baseline LVEF, mean [SD]	
HF Hospitalization in last 12 months, n (%)	
Baseline lab evaluation, mean [SD]	
Sodium, mEq/L	
Potassium, mEq/L	
Creatinine, mg/dL	
Estimated GFR, mL, min	
Brain Natriuretic Peptide, pg/mL	
Baseline vital signs, mean [SD]	
Systolic Blood Pressure, mmHg	
Diastolic Blood Pressure, mmHg	
Heart rate, bpm	
Baseline Medications*, y (%)	
Loop Diuretic	
Loop Diuretic Type	

Lasix	
Dose, mean [SD]	
Bumex	
Dose, mean [SD]	
ACE inhibitor, n (%)	
ACE inhibitor Type	
Dose, mean {SD]`	
ARB, n (%)	
ARB Type	
Dose, mean (SD)	
ARNI, n %	
Dose, mean (SD)**	
Beta-blocker	
Beta-blocker Type	
Metoprolol, y (n%)	
Dose, mean (SD)	
Carvedilol	
Dose, mean (SD)	
MRA, y (n%)	
Spironolactone, y (n)	
Dose, mean (SD)	
SGLT2 inhibitor, y (%)	
Hydralazine, y (%)	
Nitrates, y (%)	
Isosorbide Mononitrate, n (%)	
Dose, mean (SD)	
Isosorbide dinitrate, n (%)	
Dose, mean (SD)	
Digoxin, n (%)	
Device Therapy	
ICD, y (%)	
CRT-D, y (%)	

* Except loop diuretics, all rates are determined among eligible patients as detailed in methods. CAD-coronary artery disease, CKD-chronic kidney disease, HF-heart failure, LVEF-left ventricular ejection fraction, GFR-glomerular filtration rate, ACE- angiotensin converting enzyme, ARB- angiotensin receptor blocker, ARNI- Angiotensin Receptor-Neprilysin Inhibitor, SGLT2-sodium glucose co-transporter 2, MRA-Mineralocorticoid Receptor Antagonist , SGLT2-*dose of Valsartan component
