# Supplementary material for: Telephone-Based Guideline-Directed Medical Therapy Optimization in Navajo Nation: The Hózhó Randomized Clinical Trial
Source: JAMA Intern Med. 2024 Apr 7;184(6):681–90. doi: 10.1001/jamainternmed.2024.1523 (PMC11000136; doi:10.1001/jamainternmed.2024.1523)
Supplement: Supplement 2. — eFigure 1. Map of Trial Sites and Setting eFigure 2. Flowcharts/Protocols for Telehealth Model eTable 1. Eligibility/Exclusion Criteria for Each GDMT Therapy eTable 2. Number of Guideline-Directed Medical Therapies by Cohort at Baseline eTable 3. Multivariable Analyses of the Association of Intervention (Telehealth Model) With the Primary Outcome (Addition of GDMT Class at 30 Days) eTable 4. Multivariable Analyses of the Association of Intervention (Telehealth Model) With the Secondary Outcome of Addition of or Increase in Dose of GDMT eFigure 3. Spaghetti Plot of Secondary Outcome (Addition of GDMT Drug Class or Increase in GDMT Drug Dose) Over Time by Cohort eFigure 4. Spaghetti Plot of ACEi/ARB/ARNI Addition Over Time by Cohort eFigure 5. Spaghetti Plot of β-Blocker Addition Over Time by Cohort eFigure 6. Spaghetti Plot of MRA Addition Over Time by Cohort eFigure 7. Spaghetti Plot of SGLT2i Addition Over Time by Cohort eFigure 8. Spaghetti Plot of Addition or Increase in Dose of ACEi/ARB/ARNI Over Time by Cohort eFigure 9. Spaghetti Plot of Addition or Increase in Dose of β-Blocker Over Time by Cohort eFigure 10. Spaghetti Plot of Addition or Increase in Dose of MRA Over Time by Cohort eTable 5. Longer-Term GDMT Rates for Cohorts 1-3 eTable 6. Cardiac Procedures/Interventions by Study Arm eTable 7. Adverse Events Over Time for the Cohort eMethods. Additional Statistical Analysis Details on Power Analysis, Delta Method Used to Derive Confidence Intervals, and Success Rates [file jamainternmed-e241523-s002.pdf]

Supplemental Online Content

Eberly LA, Tennison A, Mays D, et al. Telephone-based guideline-directed medical therapy optimization in Navajo Nation: the Hózhó randomized clinical trial. *JAMA Intern Med*. Published online April 7, 2024. doi:10.1001/jamainternmed.2024.1523

eFigure 1. Map of Trial Sites and Setting
eFigure 2. Flowcharts/Protocols for Telehealth Model
eTable 1. Eligibility/Exclusion Criteria for Each GDMT Therapy
eTable 2. Number of Guideline-Directed Medical Therapies by Cohort at Baseline
eTable 3. Multivariable Analyses of the Association of Intervention (Telehealth Model) With the Primary Outcome (Addition of GDMT Class at 30 Days)
eTable 4. Multivariable Analyses of the Association of Intervention (Telehealth Model) With the Secondary Outcome of Addition of or Increase in Dose of GDMT
eFigure 3. Spaghetti Plot of Secondary Outcome (Addition of GDMT Drug Class or Increase in GDMT Drug Dose) Over Time by Cohort
eFigure 4. Spaghetti Plot of ACEi/ARB/ARNI Addition Over Time by Cohort
eFigure 5. Spaghetti Plot of β-Blocker Addition Over Time by Cohort
eFigure 6. Spaghetti Plot of MRA Addition Over Time by Cohort
eFigure 7. Spaghetti Plot of SGLT2i Addition Over Time by Cohort
eFigure 8. Spaghetti Plot of Addition or Increase in Dose of ACEi/ARB/ARNI Over Time by Cohort
eFigure 9. Spaghetti Plot of Addition or Increase in Dose of β-Blocker Over Time by Cohort
eFigure 10. Spaghetti Plot of Addition or Increase in Dose of MRA Over Time by Cohort
eTable 5. Longer-Term GDMT Rates for Cohorts 1-3
eTable 6. Cardiac Procedures/Interventions by Study Arm
eTable 7. Adverse Events Over Time for the Cohort
eMethods. Additional Statistical Analysis Details on Power Analysis, Delta Method Used to Derive Confidence Intervals, and Success Rates

This supplementary material has been provided by the authors to give readers additional information about their work.

© 2024 Eberly LA et al. *JAMA Internal Medicine*.

eFigure 1. Map of Trial Sites and Setting

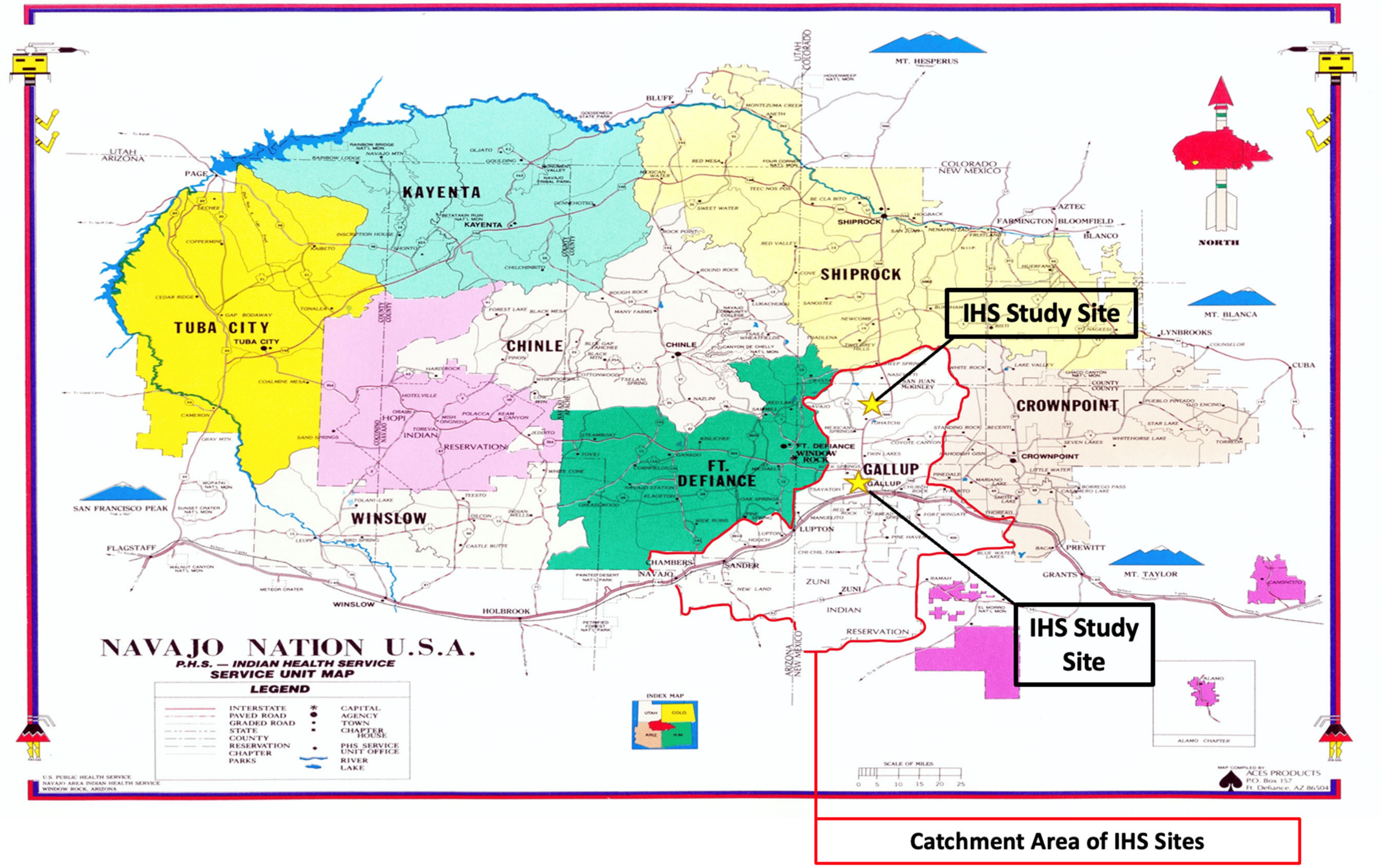

Adapted from Navajo Land Department, Division of Natural Resources: Judicial Districts of Navajo Nation. https://courts.navajo-nsn.gov/districts.htm
Map depicts communities and chapters of the Navajo Nation and location of the two IHS study sites in Eastern Navajo Nation. The catchment area (communities/chapters served by the two IHS study sites) is outlined in red. IHS-Indian Health Service.

© 2024 Eberly LA et al. *JAMA Internal Medicine*.

eFigure 2. Flowcharts/Protocols for Telehealth Model

2.1 Telementoring Model Protocol

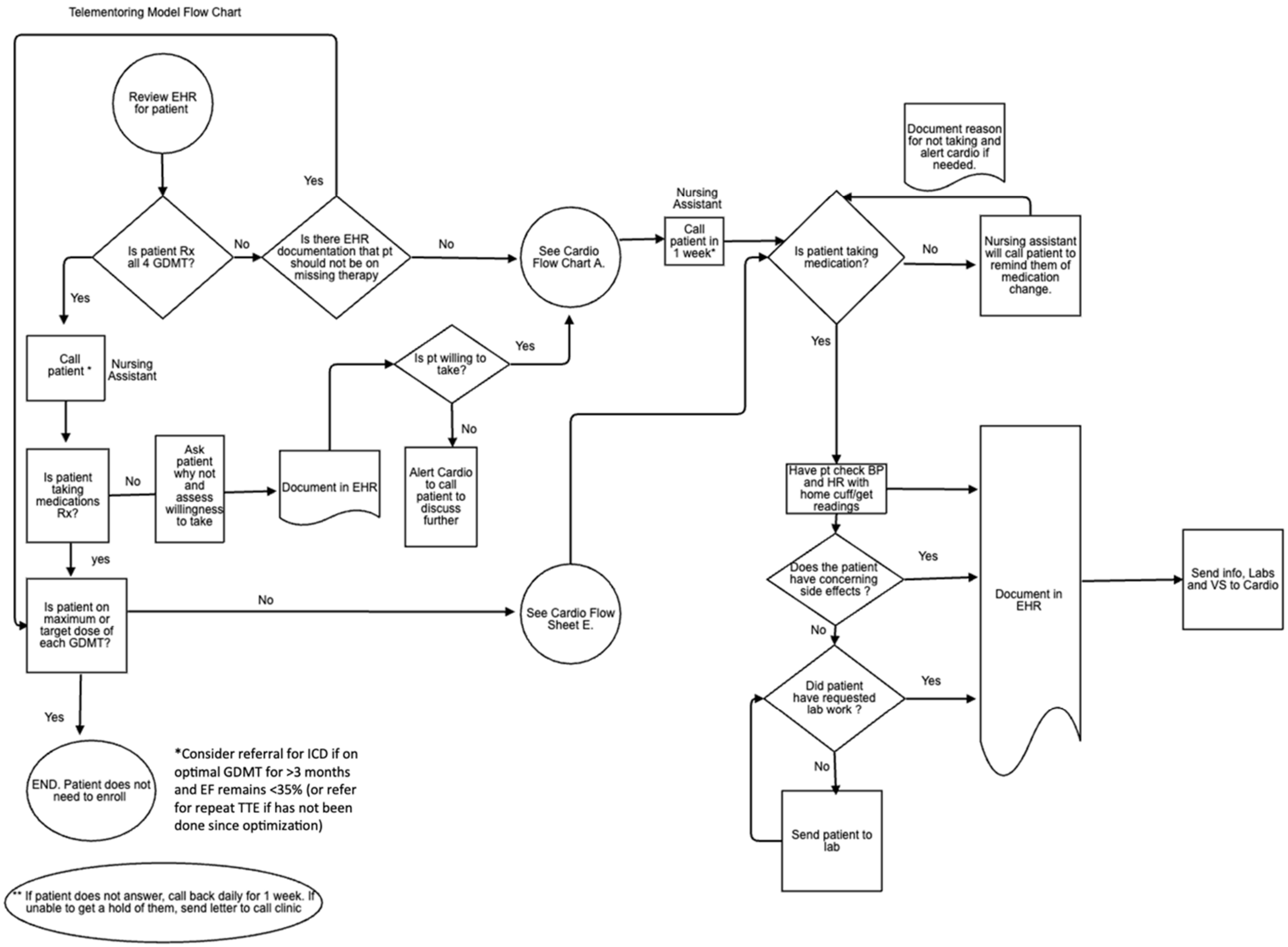

© 2024 Eberly LA et al. *JAMA Internal Medicine*.

2.2 Cardio Flow Chart A: Start + Beta-blocker

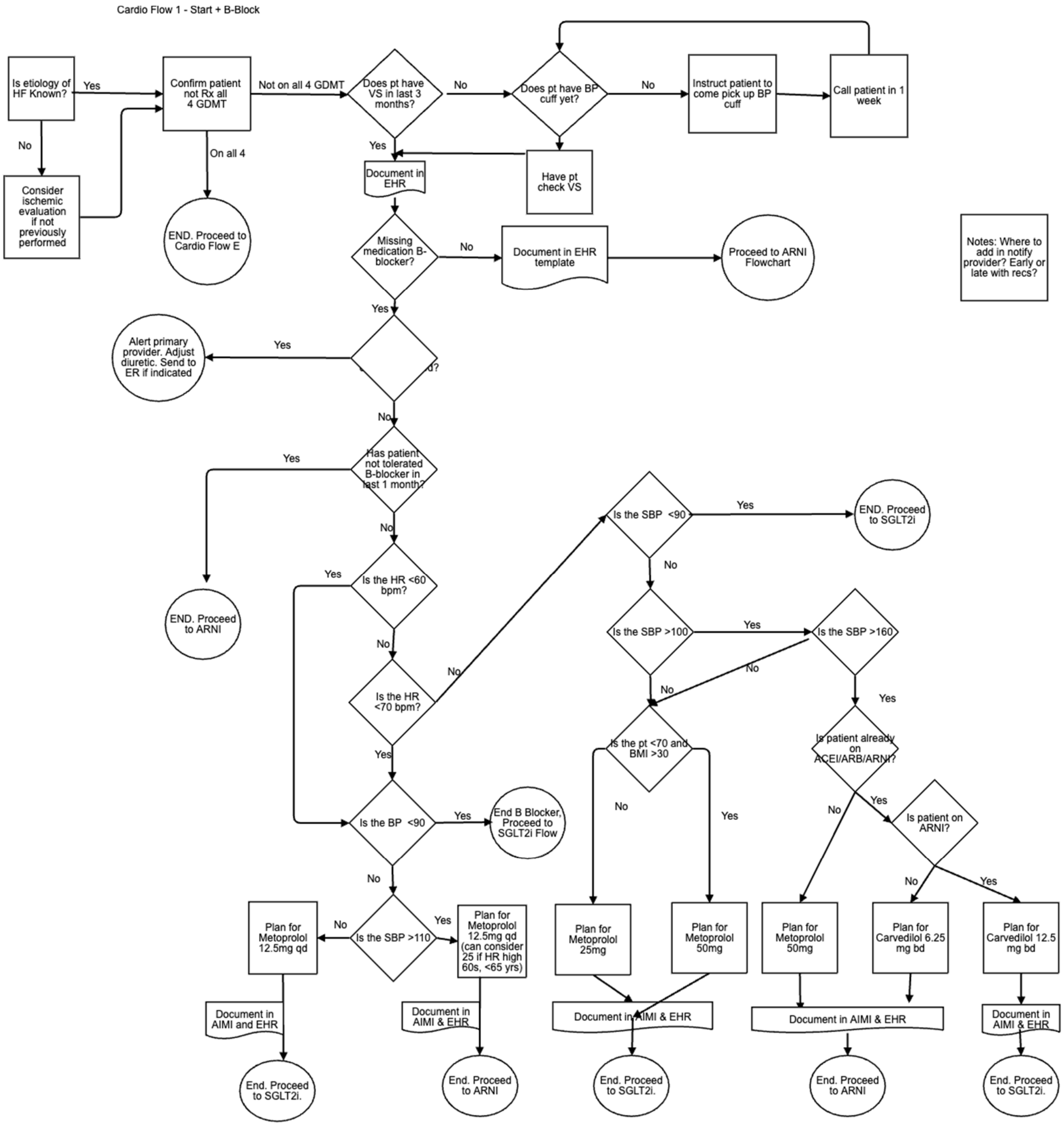

© 2024 Eberly LA et al. *JAMA Internal Medicine*.

2.3 Cardio Flow Chart B: ARNI

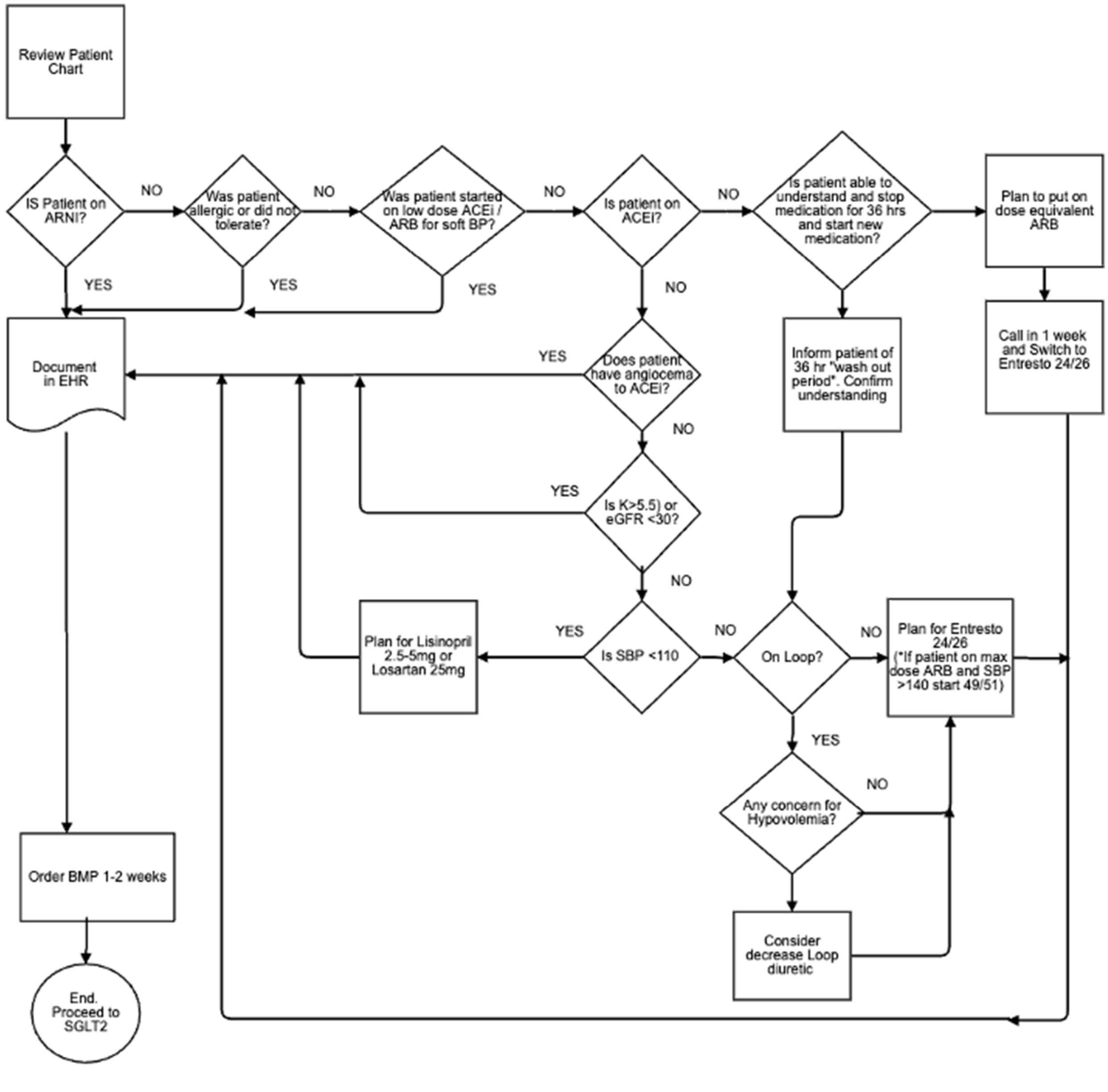

© 2024 Eberly LA et al. *JAMA Internal Medicine*.

2.4 Cardio Flow C: SGLT2i

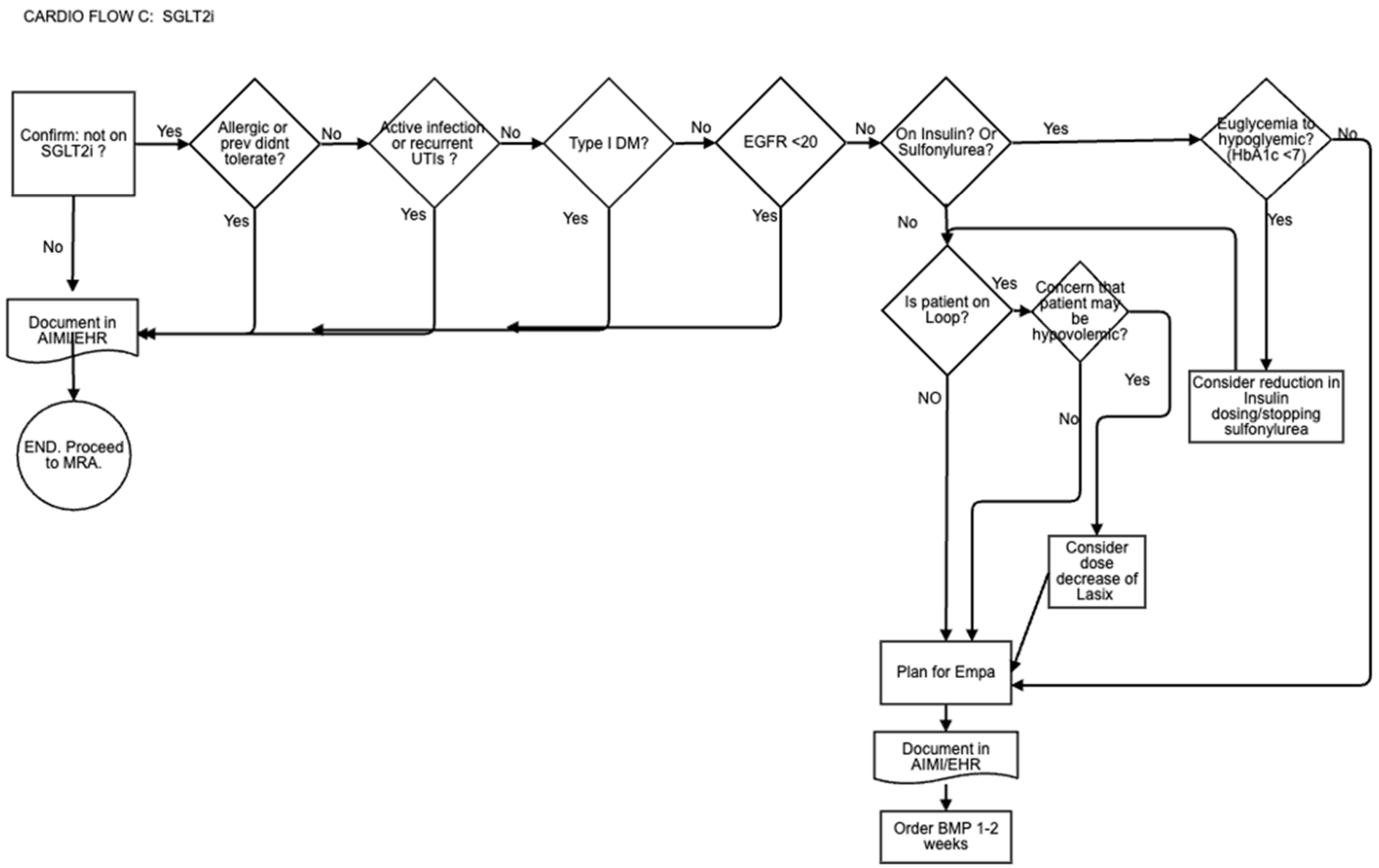

© 2024 Eberly LA et al. *JAMA Internal Medicine*.

2.5 Cardio Flow D: MRA

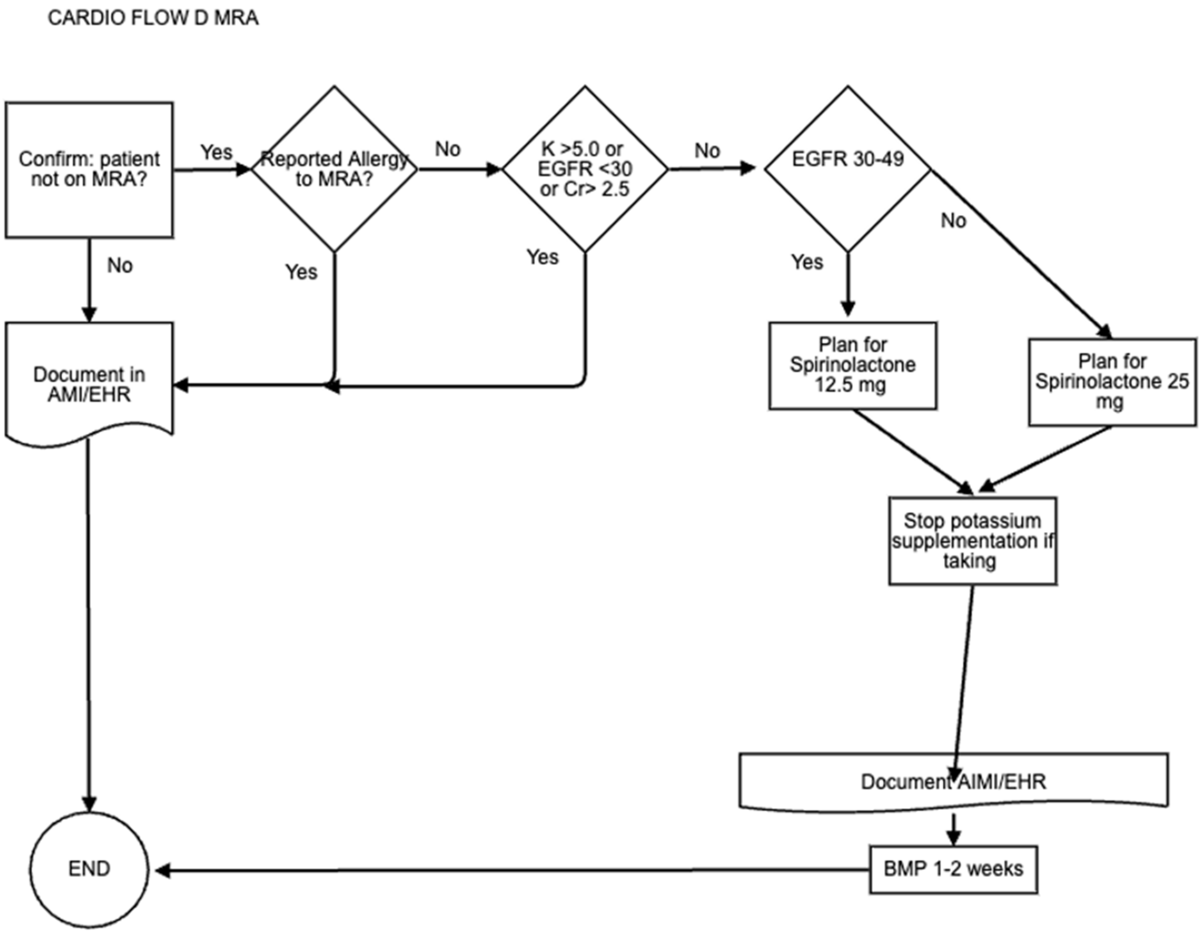

© 2024 Eberly LA et al. *JAMA Internal Medicine*.

2.6 Cardio Flow E. GDMT Titration

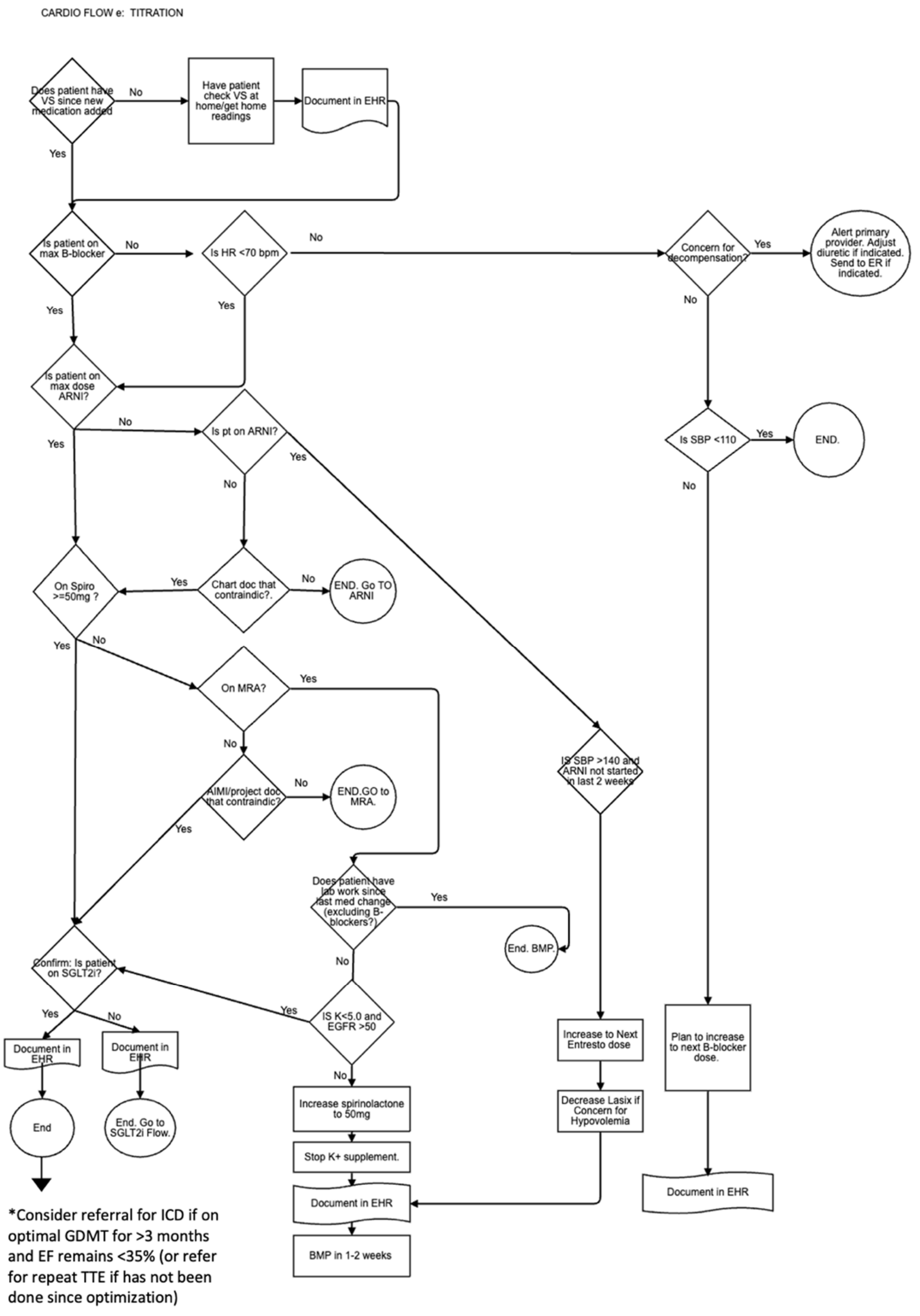

© 2024 Eberly LA et al. *JAMA Internal Medicine*.

eTable 1. Eligibility/Exclusion Criteria for Each GDMT Therapy

	BB	ACEi/ARB	ARNI	MRA	SGLT2i
Allergy to Therapy	X	X	X	X	X
Previously did not tolerate (hypotension, hyperkalemia, etc)	X	X	X	X	X
Most recent systolic BP <100 mmHg	X	X	X	X	
Most recent Potassium >5 mEq/L		X	X	X	
Most Recent Creatinine >2.5 mg/dL or GFR <30 (not on dialysis)		X	X	X	* SGLT2i excluded only at GFR <20
Hemodialysis				X	X
Frequent complicated UTIs					X
Type I DM					X

© 2024 Eberly LA et al. *JAMA Internal Medicine*.

eTable 2. Number of Guideline-Directed Medical Therapies by Cohort at Baseline

Supplemental Table 2.1. Number of Guideline-directed Medical Therapies by Cohort at Baseline with Denominator as all patients (n=103)

	Cohort 1	Cohort 2	Cohort 3	Cohort 4	Cohort 5
On 0 therapy	0/103 (0%)	0/103 (0%)	0/103 (0%)	0/103 (0%)	0/103 (0%)
On 1 therapy	2/103 (1.9%)	2/103 (1.9%)	3/103 (2.9%)	2/103 (1.9%)	5/103 (4.9%)
On 2 therapies	3/103 (2.9%)	7/103 (6.8%)	5/103 (4.9%)	10/103 (9.7%)	7/103 (6.8%)
On 3 therapies	10/103 (9.7%)	6/103 (5.8%)	9/103 (8.7%)	4/103 (3.9%)	4/103 (3.9%)
On 4 therapies	6/103 (5.8%)	6/103 (5.8%)	3/103 (2.9%)	4/103 (3.9%)	5/103 (4.9%)

*Therapy categories include Beta-blocker, ACEI/ARB/ARNI, SGLT2i, or MRA.

Supplemental Table 2.2. Number of Guideline-directed Medical Therapies by Cohort at Baseline with Denominator as Patients that are Eligible for the Therapy

	Cohort 1	Cohort 2	Cohort 3	Cohort 4	Cohort 5
On 0 therapy	0/0 (NA)	0/0 (NA)	0/0 (NA)	0/0 (NA)	0/0 (NA)
On 1 therapy	2/0 (NA)	2/0 (NA)	3/0 (NA)	2/0 (NA)	5/0 (NA)
On 2 therapies	3/10 (30%)	7/10 (70%)	5/10 (50%)	10/10 (100%)	7/10 (70%)
On 3 therapies	10/11 (90.9%)	6/11 (54.5%)	9/11 (81.8%)	4/11 (36.4%)	4/11 (36.4%)
On 4 therapies	6/82 (7.3%)	6/82 (7.3%)	3/82 (3.7%)	4/82 (4.9%)	5/82 (6.1%)

*Therapy categories include Beta-blocker, ACEI/ARB/ARNI, SGLT2i, or MRA.

© 2024 Eberly LA et al. *JAMA Internal Medicine*.

eTable 3. Multivariable Analyses of the Association of Intervention (Telehealth Model) With the Primary Outcome (Addition of GDMT Class at 30 Days)

	OR (95% CI)	p-value
**Intervention (Telehealth Model)**	26.39 (10.20-68.28)	<0.001
**Age**	1.19 (0.94-1.52)	0.149
**Male Sex**	1.44 (0.71-2.93)	0.309
**LVEF**	0.96 (0.91-1.00)	0.06
**Coronary Artery Disease**	0.75 (0.36-1.55)	0.435
**Diabetes**	2.27 (1.02-5.05)	0.045
**GDMT Classes at Baseline**	0.26 (0.16-0.42)	<0.001

LVEF-left ventricular ejection fraction; GDMT-guideline-directed medical therapy.

© 2024 Eberly LA et al. *JAMA Internal Medicine*.

eTable 4. Multivariable Analyses of the Association of Intervention (Telehealth Model) With the Secondary Outcome of Addition of or Increase in Dose of GDMT

	**OR (95% CI)**	**p-value**
**Intervention (Telehealth Model)**	18.00 (8.85-36.60)	<0.001
**Age**	0.89 (0.70-1.13)	0.330
**Male Sex**	1.17 (0.61-2.24)	0.641
**LVEF**	0.95 (0.91-0.99)	0.01
**Coronary Artery Disease**	1.92 (0.98-3.73)	0.056
**Diabetes**	2.48 (1.20-5.10)	0.014
**GDMT Classes at Baseline**	0.51 (0.33-0.79)	0.003

LVEF-left ventricular ejection fraction; GDMT-guideline-directed medical therapy.

© 2024 Eberly LA et al. *JAMA Internal Medicine*.

eFigure 3. Spaghetti Plot of Secondary Outcome (Addition of GDMT Drug Class or Increase in GDMT Drug Dose) Over Time by Cohort

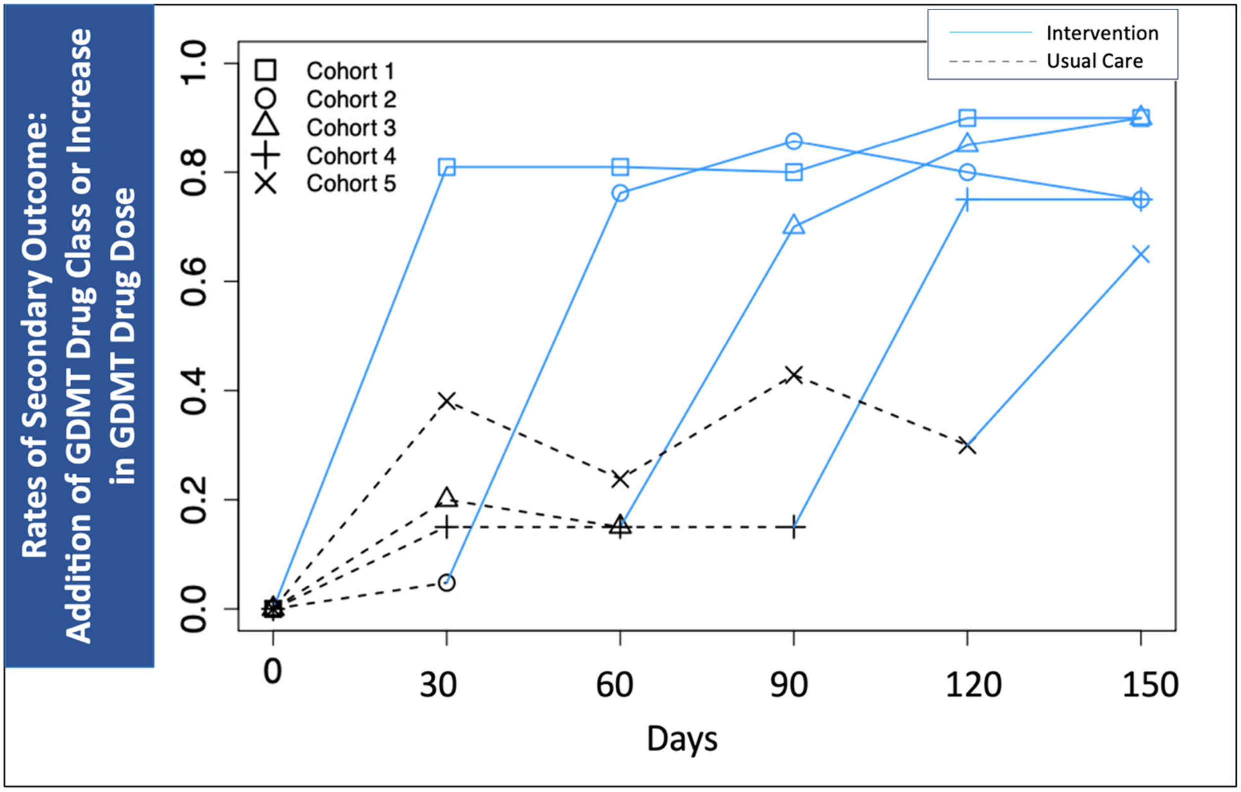

Cluster 1 had immediate implementation of the telehealth model, cluster 2 at 30 days, cluster 3 at 60 days, cluster 4 at 90 days, and cluster 5 at 120 days. The blue line represents time when a cohort is in the intervention arm, and black when the cohort is under usual care. As demonstrated, there was a significant and rapid increase in number of guideline-directed medical therapy classes and doses and rates of the secondary outcome as clusters crossed over into the intervention. GDMT-guideline directed medical therapy.

© 2024 Eberly LA et al. *JAMA Internal Medicine*.

eFigure 4. Spaghetti Plot of ACEi/ARB/ARNI Addition Over Time by Cohort

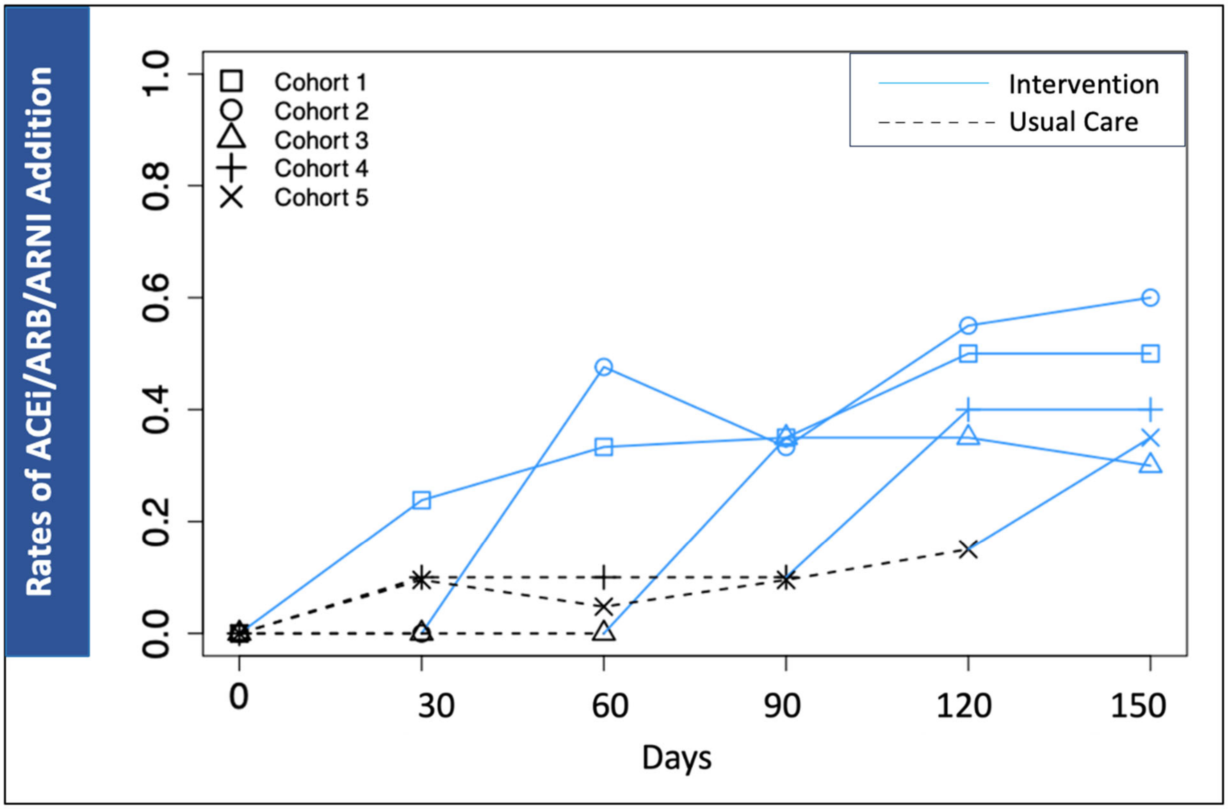

Cluster 1 had immediate implementation of the telehealth model, cluster 2 at 30 days, cluster 3 at 60 days, cluster 4 at 90 days, and cluster 5 at 120 days. The blue line represents time when a cohort is in the intervention arm, and black when the cohort is under usual care. As demonstrated, there was a significant and rapid increase in ACEi/ARB/ARNI therapy as clusters crossed over into the intervention. ACEi-angiotensin-converting enzyme inhibitor; ARB-angiotensin receptor blocker; ARNI-angiotensin receptor-neprilysin inhibitor.

© 2024 Eberly LA et al. *JAMA Internal Medicine*.

eFigure 5. Spaghetti Plot of β-Blocker Addition Over Time by Cohort

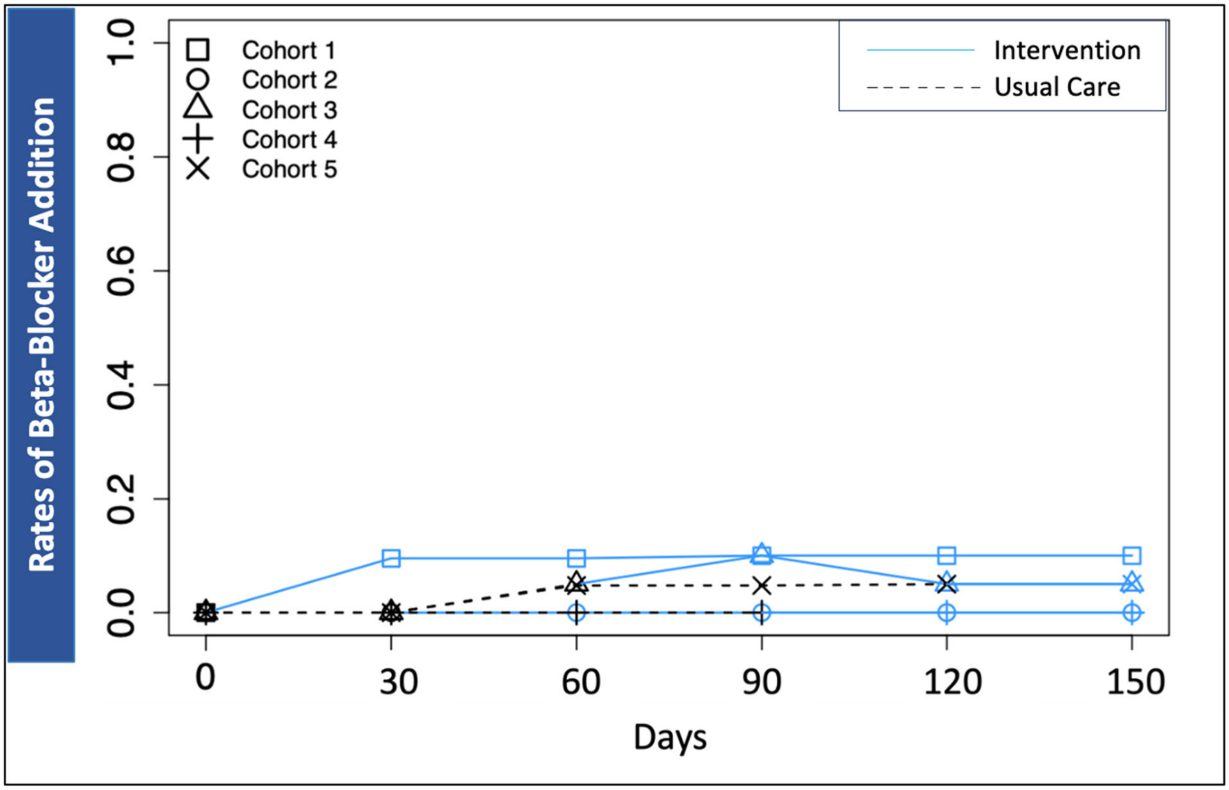

Cluster 1 had immediate implementation of the telehealth model, cluster 2 at 30 days, cluster 3 at 60 days, cluster 4 at 90 days, and cluster 5 at 120 days. The blue line represents time when a cohort is in the intervention arm, and black when the cohort is under usual care. There was no statistically significant increase in beta-blocker therapy between study arms.

© 2024 Eberly LA et al. *JAMA Internal Medicine*.

eFigure 6. Spaghetti Plot of MRA Addition Over Time by Cohort

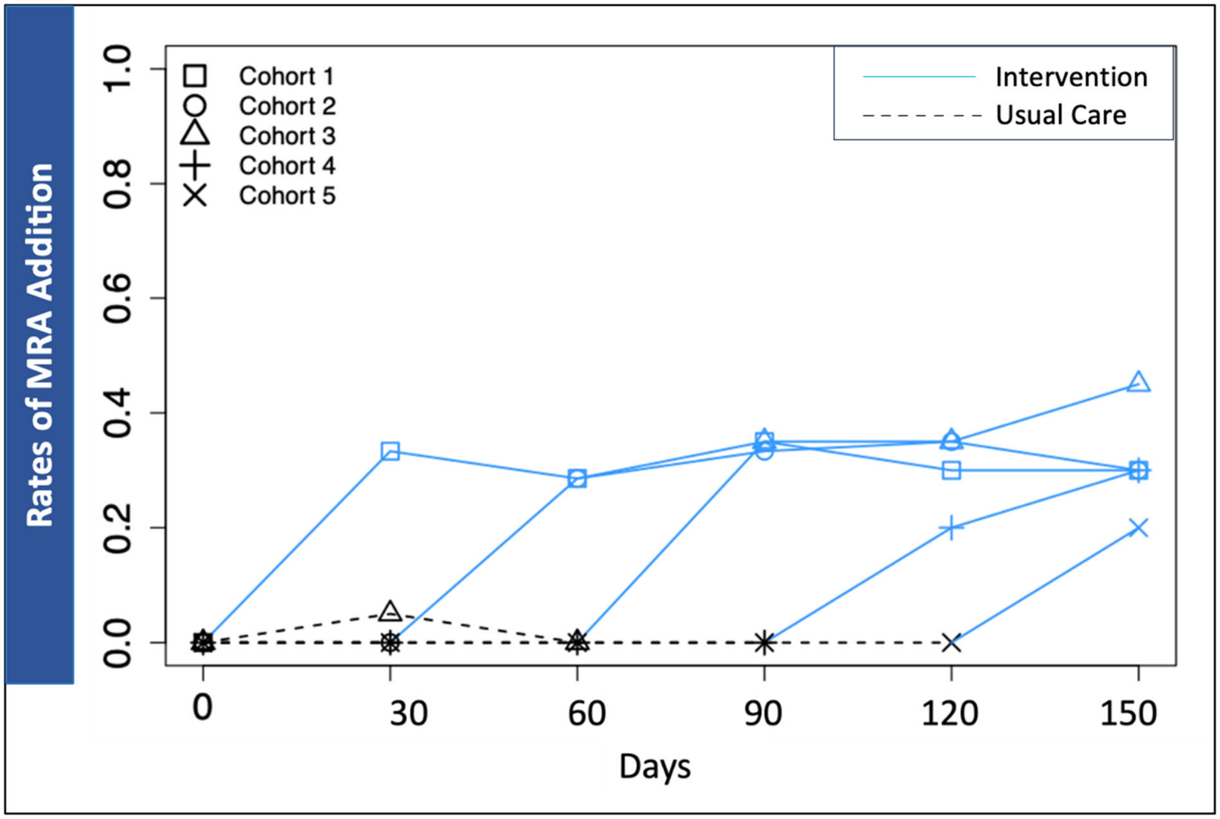

Cluster 1 had immediate implementation of the telehealth model, cluster 2 at 30 days, cluster 3 at 60 days, cluster 4 at 90 days, and cluster 5 at 120 days. The blue line represents time when a cohort is in the intervention arm, and black when the cohort is under usual care. As demonstrated, there was a significant and rapid increase in MRA therapy as clusters crossed over into the intervention. MRA-mineralocorticoid receptor antagonist.

© 2024 Eberly LA et al. *JAMA Internal Medicine*.

eFigure 7. Spaghetti Plot of SGLT2i Addition Over Time by Cohort

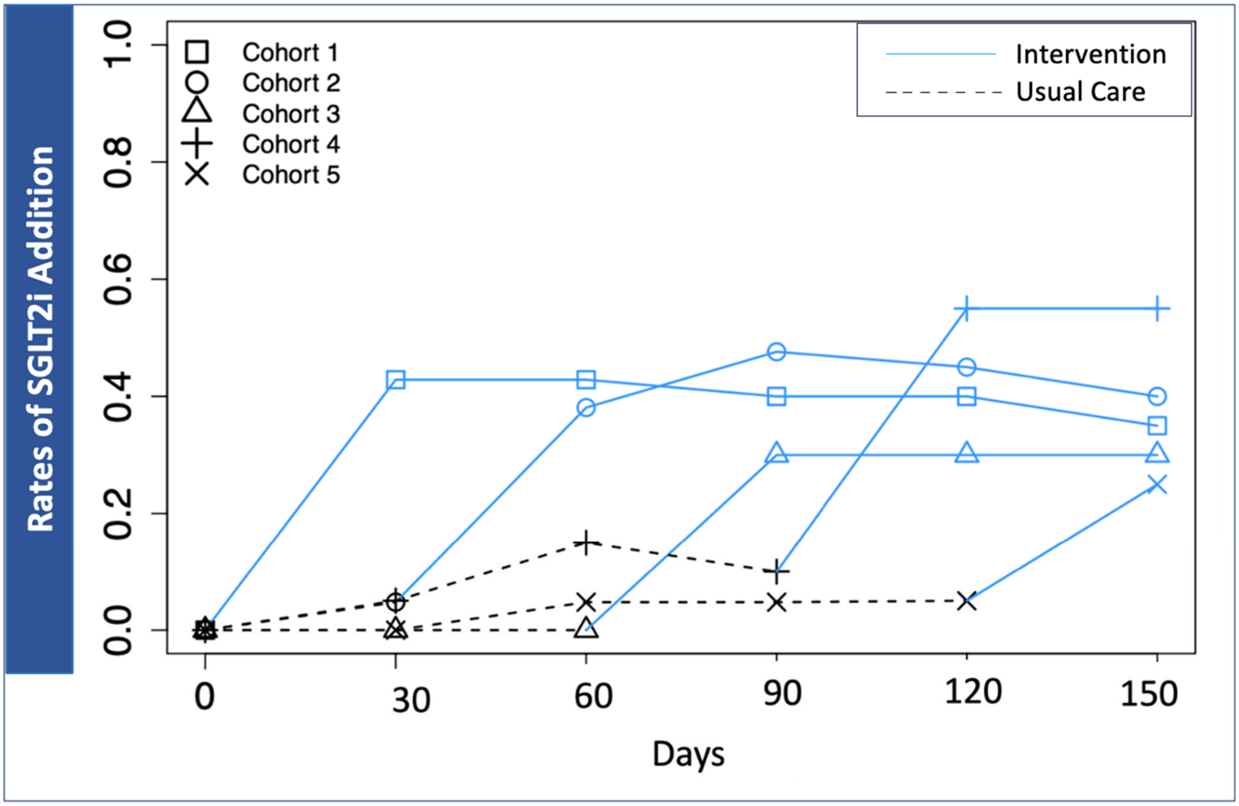

Cluster 1 had immediate implementation of the telehealth model, cluster 2 at 30 days, cluster 3 at 60 days, cluster 4 at 90 days, and cluster 5 at 120 days. The blue line represents time when a cohort is in the intervention arm, and black when the cohort is under usual care. As demonstrated, there was a significant and rapid increase in SGLT2i therapy as clusters crossed over into the intervention. SGLT2i- sodium-glucose cotransporter-2 inhibitor.

© 2024 Eberly LA et al. *JAMA Internal Medicine*.

eFigure 8. Spaghetti Plot of Addition or Increase in Dose of ACEi/ARB/ARNI Over Time by Cohort

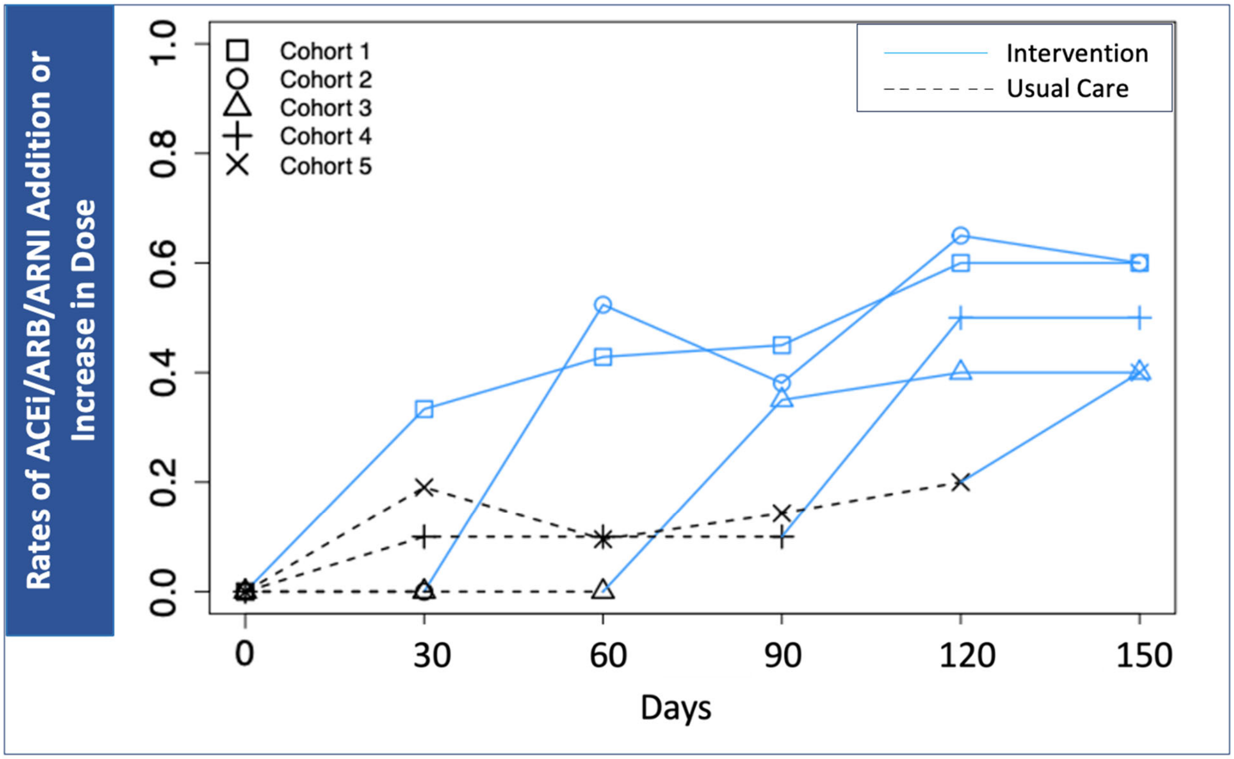

Cluster 1 had immediate implementation of the telehealth model, cluster 2 at 30 days, cluster 3 at 60 days, cluster 4 at 90 days, and cluster 5 at 120 days. The blue line represents time when a cohort is in the intervention arm, and black when the cohort is under usual care. ACEi- angiotensin-converting enzyme inhibitor; ARB-angiotensin receptor blocker; ARNI-angiotensin receptor-neprilysin inhibitor.

© 2024 Eberly LA et al. *JAMA Internal Medicine*.

eFigure 9. Spaghetti Plot of Addition or Increase in Dose of β-Blocker Over Time by Cohort

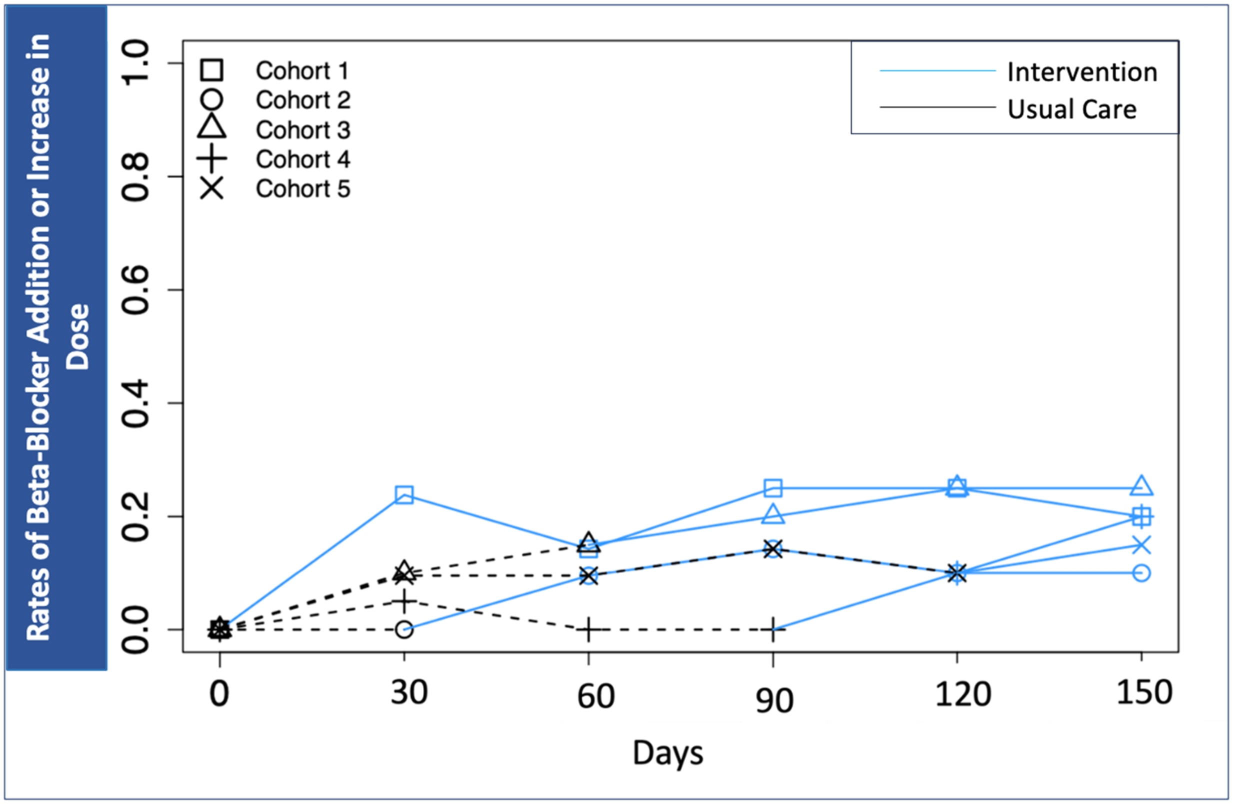

Cluster 1 had immediate implementation of the telehealth model, cluster 2 at 30 days, cluster 3 at 60 days, cluster 4 at 90 days, and cluster 5 at 120 days. The blue line represents time when a cohort is in the intervention arm, and black when the cohort is under usual care.

© 2024 Eberly LA et al. *JAMA Internal Medicine*.

eFigure 10. Spaghetti Plot of Addition or Increase in Dose of MRA Over Time by Cohort

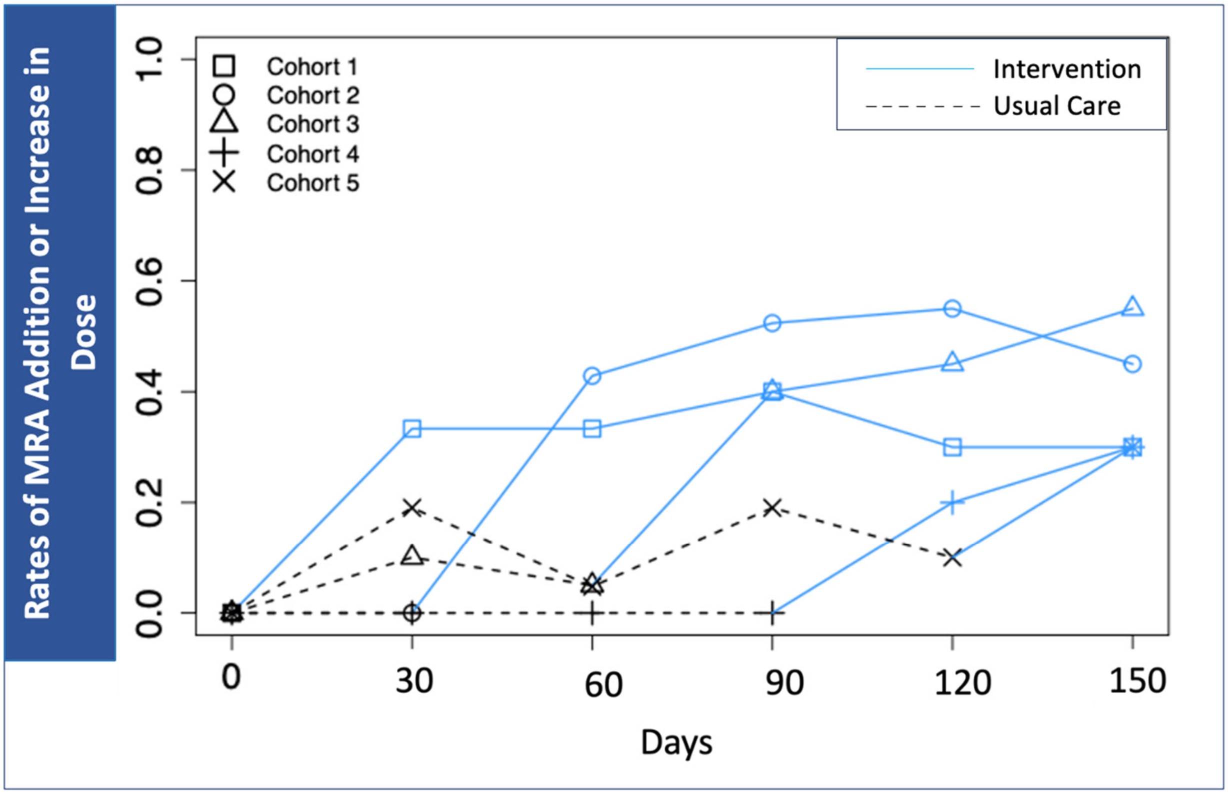

Cluster 1 had immediate implementation of the telehealth model, cluster 2 at 30 days, cluster 3 at 60 days, cluster 4 at 90 days, and cluster 5 at 120 days. The blue line represents time when a cohort is in the intervention arm, and black when the cohort is under usual care. MRA-mineralocorticoid receptor antagonist.

© 2024 Eberly LA et al. *JAMA Internal Medicine*.

eTable 5. Longer-Term GDMT Rates for Cohorts 1-3

Supplemental Table 5.1. Proportion of patients with the use of each therapy in cohort 1 at 5 months

	Cohort 1
ACEi, ARB or ARNI	20/20 (100%)
B blocker	20/20 (100%)
SGTL2	18/20 (90%)
MRA	18/20 (90%)

Supplemental Table 5.2. Proportion of patients with the use of each therapy in cohort 2 and 3 at 3 months

	Cohort 2	Cohort 3
ACEi, ARB or ARNI	18/21 (85.7%)	19/20 (95%)
B blocker	21/21 (100%)	20/20 (100%)
SGTL2	20/21 (95.2%)	16/20 (80%)
MRA	16/21 (76.2%)	13/20 (65%)

© 2024 Eberly LA et al. *JAMA Internal Medicine*.

eTable 6. Cardiac Procedures/Interventions by Study Arm

	**Intervention**	**Usual Care**	**p-value**
**Total Cardiac Procedures/Interventions**	7 (5%)	1 (2.3%)	0.15
Types of Cardiac Procedures			
Coronary Revascularization	4 (2 coronary artery bypass graft surgery, 2 multivessel percutaneous coronary intervention	0	
CRT-D	2	0	
ICD	1	0	
TAVR	0	1	

CRT-D- Cardiac Resynchronization Therapy Defibrillator (CRT-D); ICD-implantable Cardioverter Defibrillator; TAVR-Transcatheter aortic valve replacement

© 2024 Eberly LA et al. *JAMA Internal Medicine*.

eTable 7. Adverse Events Over Time for the Cohort

Adverse Events	Intervention	1 month	2 months	3 months	4 months	5 months
Hypotension						
	No	1/82 (1.2%)	1/61 (1.6%)	0/41 (0%)	0/21 (0%)	NA
	Yes	1/21 (4.8%)	1/42 (2.4%)	0/61 (0%)	2/80 (2.5%)	2/100 (2%)
UTI						
	No	0/82 (0%)	0/61 (0%)	1/41 (2.4%)	0/21 (0%)	NA
	Yes	0/21 (0%)	1/42 (2.4%)	0/61 (0%)	1/80 (1.2%)	2/100 (2.0%)
Hyperkalemia						
	No	0/82 (0%)	0/61 (0%)	0/41 (0%)	0/21 (0%)	NA
	Yes	0/21 (0%)	0/42 (0%)	0/61 (0%)	3/80 (3.8%)	0/100 (0%)
Hypokalemia						
	No	0/82 (0%)	0/61(0%)	0/41 (0%)	0/21 (0%)	NA
	Yes	1/21 (4.8%)	1/42 (2.4%)	0/61 (0%)	0/80 (0%)	0/100 (0%)
Yeast Infection						
	No	0/82 (0%)	0/61 (0%)	0/41 (0%)	0/21 (0%)	NA
	Yes	0/21 (0%)	0/42 (0%)	0/61 (0%)	1/80 (1.2%)	0/100 (0%)
AKI						
	No	1/82 (1.2%)	1/61 (1.6%)	1/41 (2.4%)	0/21 (0%)	NA
	Yes	0/21 (0%)	0/42 (0%)	0/61 (0%)	2/80 (2.5%)	0/100 (0%)
Hyponatremia						
	No	1/82 (1.2%)	0/61 (0%)	0/41 (0%)	0/21 (0%)	NA
	Yes	0/21 (0%)	0/42 (0%)	0/61 (0%)	0/80 (0%)	0/100 (0%)
Death						
	No	0/82 (0%)	0/61 (0%)	0/41 (0%)	1/21 (4.8%)	NA
	Yes	0/21 (0%)	0/42 (0%)	0/61 (0%)	0/80 (0%)	0/100 (0%)
Volume Overload						
	No	0/82 (0%)	0/61 (0%)	0/41 (0%)	3/21 (14.3%)	NA
	Yes	0/21 (0%)	0/42 (0%)	0/61 (0%)	0/80 (0%)	2/100 (2%)
ER visit						
	No	0/82 (0%)	0/61 (0%)	0/41 (0%)	1/21 (4.8%)	NA
	Yes	0/21 (0%)	0/42 (0%)	0/61 (0%)	0/80 (0%)	3/100 (3%)

UTI-urinary tract infection; AKI-acute kidney injury; ER- emergency room.

© 2024 Eberly LA et al. *JAMA Internal Medicine*.

Supplemental Table 7.1. Total Adverse Events

	No intervention	Intervention
1_months	3/82 (3.7%)	2/21 (9.5%)
2_months	1/61 (1.6%)	3/42 (7.1%)
3_months	2/41 (4.9%)	0/61 (0.0%)
4_months	4/21 (19.0%)	7/80 (8.8%)
5_months	NA/NA (NA%)	8/100 (8.0%)

Supplemental Table 7.2. Volume Overload

	No intervention	Intervention
1_months	0/82 (0.0%)	0/21 (0.0%)
2_months	0/61 (0.0%)	0/42 (0.0%)
3_months	0/41 (0.0%)	0/61 (0.0%)
4_months	2/21 (9.5%)	0/78 (0.0%)
5_months	NA/NA (NA%)	1/100 (1.0%)

Supplemental Table 7.3 Hyperkalemia

	No intervention	Intervention
1_months	0/82 (0.0%)	0/21 (0.0%)
2_months	0/61 (0.0%)	0/42 (0.0%)
3_months	0/41 (0.0%)	0/61 (0.0%)
4_months	0/21 (0.0%)	1/78 (1.3%)
5_months	NA/NA (NA%)	0/100 (0.0%)

Supplemental Table 7.4. Hypokalemia

	No intervention	Intervention
1_months	0/82 (0.0%)	1/21 (4.8%)
2_months	0/61 (0.0%)	1/42 (2.4%)
3_months	0/41 (0.0%)	0/61 (0.0%)
4_months	0/21 (0.0%)	0/78 (0.0%)
5_months	NA/NA (NA%)	0/100 (0.0%)

Supplemental Table 7.5. Acute Kidney Injury and Hypotension

	No intervention	Intervention
1_months	2/82 (2.4%)	1/21 (4.8%)
2_months	1/61 (1.6%)	1/42 (2.4%)
3_months	1/41 (2.4%)	0/61 (0.0%)
4_months	0/21 (0.0%)	2/78 (2.6%)
5_months	NA/NA (NA%)	2/100 (2.0%)

© 2024 Eberly LA et al. *JAMA Internal Medicine*.

eMethods. Additional Statistical Analysis Details on Power Analysis, Delta Method Used to Derive Confidence Intervals, and Success Rates

Details for Power Analysis:

Step 1. Data generation. 100 participants are divided into 5 groups, 20 participants per group. Generate 5 binary outcomes for each participant. The success rates for the 5 binary outcomes in group 1 are $(p_1, p_1, p_1, p_1, p_1)$; in group 2 are $(p_0, p_1, p_1, p_1, p_1)$; in group 3 are $(p_0, p_0, p_1, p_1, p_1)$; in group 4 are $(p_0, p_0, p_0, p_1, p_1)$; in group 5 are $(p_0, p_0, p_0, p_0, p_1)$, where $p_1 = 0.35$ and $p_0 = 0.10$, and ICC = 0.025 within participants.

Step 2. Data analysis. Perform a logistic regression model with the GEE on the simulated data and determine if the log OR estimate is significant at a significance level of 0.05.

Step 3. Simulation replication. Repeat Steps 1 and 2 10,000 times and calculate the proportion of significant log OR estimates among the 10,000 simulation replications. The proportion is the empirical power.

Because the true ICC is unknow, we tried various ICC values: 0.025, 0.2, 0.4, and 0.6. The simulation results show the empirical powers at these ICC values can achieve at least 80%. To be conservative, we chose ICC=0.025 due to its smaller empirical power compared with those using the other ICC values.

© 2024 Eberly LA et al. *JAMA Internal Medicine*.

Details of the Delta Method:

Assume

$$\sqrt{n}(\widehat{\beta} - \beta) \rightarrow N(0, \Sigma_\beta) \text{ in distribution.}$$

The delta method implies, for a function g that is differentiable with respect to β,

$$\sqrt{n}(g(\widehat{\beta}) - g(\beta)) \rightarrow N\left(0, \nabla g(\beta)^T \, \Sigma_\beta \, \nabla g(\beta)\right) \text{ in distribution,}$$

where $\nabla g(\beta) = \left(\frac{\partial g}{\beta_0}, \frac{\partial g}{\partial \beta_1}, \dots, \frac{\partial g}{\partial \beta_p}\right)^T$. In our logistic regression case

$$\log\left(\frac{p}{1-p}\right) = \beta_0 + \beta_1 x,$$

$\widehat{\beta}$ that is estimated by GEE converges to a normal distribution. The success rate $p = g(\beta) = \exp(\beta_0 + \beta_1 x)/[1 + \exp(\beta_0 + \beta_1 x)]$, and $\nabla g(\beta) = \left(\frac{\exp(\beta_0+\beta_1 x)}{[1+\exp(\beta_0+\beta_1 x)]^2}, \frac{\exp(\beta_0+\beta_1 x)x}{[1+\exp(\beta_0+\beta_1 x)]^2}\right)^T$. By the delta method, the asymptotic variances for $\widehat{p_0} = \exp(\widehat{\beta_0})/[1 + \exp(\widehat{\beta_0})]$ and $\widehat{p_1} = \exp(\widehat{\beta_0} + \widehat{\beta_1})/[1 + \exp(\widehat{\beta_0} + \widehat{\beta_1})]$ can be obtained from the formula $\nabla g(\hat{\beta})^T \, \widehat{\Sigma_\beta} \, \nabla g(\hat{\beta})/n$ with $x = 0$ and 1, respectively. Denote the variance estimates by $\widehat{Var(\widehat{p_0})}$ and $\widehat{Var(\widehat{p_1})}$. Then, the 95% CIs for p_0 and p_1 are thus derived by $\widehat{p_x} \mp 1.96\sqrt{\widehat{Var(\widehat{p_x})}}$, $x = 0$ and 1.

Success Rates:

We denoted the success rate of usual care post the t-th 30-day by $p_{0t}, t = 1, 2, \dots, 4$ and the success rate of intervention post the t-th 30-day by $p_{1t}, t = 1, 2, \dots, 5$. Figure 4 shows the trend for the success rates was not significantly changed post the 1st 30-day of intervention or usual care. Thus, we assumed $p_0 = p_{01} = p_{02} = \cdots = p_{04}$ and $p_1 = p_{11} = p_{12} = \cdots = p_{15}$ to simplify the logistic regression model. That is, p_{11} and p_{01} of interest equal p_1 and p_0, respectively. In

© 2024 Eberly LA et al. *JAMA Internal Medicine*.

fact, the estimate for a time variable that is used to describe the time effect was not statistically significant when we included the time variable in the model.

Each patient had a binary outcome post each of five 30-day usual care or intervention. Patients in group 1 experienced five 30-day of intervention, where the success rates for the 5 outcomes were assumed to be all p_1; Patients in group 2 first experienced one 30-day of usual care and then four 30-day of intervention, where the success rates for the 5 outcomes were assumed to be $(p_0, p_1, p_1, p_1, p_1)$; Patients in group 3 first experienced two 30-day of usual care and then three 30-day of intervention, where the success rates for the 5 outcomes were assumed to be $(p_0, p_0, p_1, p_1, p_1)$; and groups 4 and 5 were so forth. All outcomes were used for estimating p_0 and p_1, thus the outcomes post usual care were treated as those in control.

All the analyses were performed with the use of Stata software version 15 (StataCorp) and R software version 4.3.1 (R Foundation for Statistical Computing) with R packages geepack 1.3.9 and simstudy 0.7.1.

© 2024 Eberly LA et al. *JAMA Internal Medicine*.
