# Supplementary material for: Telephone-Based Guideline-Directed Medical Therapy Optimization in Navajo Nation: The Hózhó Randomized Clinical Trial
Source: JAMA Intern Med. 2024 Apr 7;184(6):681–90. doi: 10.1001/jamainternmed.2024.1523 (PMC11000136; doi:10.1001/jamainternmed.2024.1523)
Supplement: Supplement 3. — Data Sharing Statement [file jamainternmed-e241523-s003.pdf]

Data Sharing Statement

Eberly. Telephone-Based Guideline-Directed Medical Therapy Optimization in Navajo Nation. *JAMA Intern Med*. Published April 07, 2024. doi:10.1001/jamainternmed.2024.1523

Data
Data available: No

Additional Information
Explanation for why data not available: Data are tribal data and therefore owned by Navajo Nation. Therefore, per Navajo Nation Human Research Review Board regulations, data cannot be made available. However, data can be made available upon reasonable request and if approved by Navajo Nation Human Research Review Board.
